# Evolutionary signals of selection on cognition from the great tit genome and methylome

**DOI:** 10.1038/ncomms10474

**Published:** 2016-01-25

**Authors:** Veronika N. Laine, Toni I. Gossmann, Kyle M. Schachtschneider, Colin J. Garroway, Ole Madsen, Koen J. F. Verhoeven, Victor de Jager, Hendrik-Jan Megens, Wesley C. Warren, Patrick Minx, Richard P. M. A. Crooijmans, Pádraic Corcoran, The Great Tit HapMap Consortium, Frank Adriaensen, Eduardo Belda, Andrey Bushuev, Mariusz Cichon, Anne Charmantier, Niels Dingemanse, Blandine Doligez, Tapio Eeva, Kjell Einar Erikstad, Slava Fedorov, Michaela Hau, Sabine Hille, Camilla Hinde, Bart Kempenaers, Anvar Kerimov, Milos Krist, Raivo Mand, Erik Matthysen, Reudi Nager, Claudia Norte, Markku Orell, Heinz Richner, Tore Slagsvold, Vallo Tilgar, Joost Tinbergen, Janos Torok, Barbara Tschirren, Tera Yuta, Ben C. Sheldon, Jon Slate, Kai Zeng, Kees van Oers, Marcel E. Visser, Martien A. M. Groenen

**Affiliations:** 1 Department of Animal Ecology, Netherlands Institute of Ecology (NIOO-KNAW), P.O. Box 50, 6700AB Wageningen, The Netherlands; 2 Department of Animal and Plant Sciences, University of Sheffield, Sheffield S10 2TN, UK; 3 Animal Breeding and Genomics Centre, Wageningen University, P.O. Box 338, 6700AH Wageningen, The Netherlands; 4 Department of Animal Sciences, University of Illinois, Urbana, Illinois 61801, USA; 5 Edward Grey Institute, Department of Zoology, University of Oxford, Oxford OX1 3PS, UK; 6 Department of Terrestrial Ecology, Netherlands Institute of Ecology (NIOO-KNAW), P.O. Box 50, 6700AB Wageningen, The Netherlands; 7 The Genome Institute, Washington University School of Medicine, St Louis, Missouri 63108, USA; 8 Evolutionary Ecology Group, Department of Biology, University of Antwerp, B-2020 Antwerp, Belgium.; 9 Departamento de Ciencia Animal, IGIC, Universidad Politécnica de Valencia, C/Paranimf n°1, E-46730 Gandía (Valencia), España.; 10 Faculty of Biology, Lomonosov Moscow State University, Moscow 119234, Russia.; 11 Inst. of Environmental Sciences, Jagiellonian University, Gronostajowa 7, 30-387 Kraków, Poland.; 12 CEFE-CNRS, UMR 5175, 1919, route de Mende, F34293 Montpellier Cedex 5, France.; 13 Max Planck Institute for Ornithology, Department of Behavioural Ecology & Evolutionary Genetics, Eberhard-Gwinner-Straße, House 5, 82319 Seewiesen (Starnberg), Germany.; 14 UMR CNRS 5558—LBBE, Biométrie et Biologie Évolutive, UCB Lyon 1 - Bât. Grégor Mendel, 43 bd du 11 novembre 1918, 69622 VILLEURBANNE cedex, France.; 15 Section of Ecology, Department of Biology, University of Turku, Turku 20014, Finland.; 16 Norwegian Institute for Nature Research, FRAM-High North Research Centre for Climate and the Environment, 9296 Tromsø, Norway.; 17 Department of Vertebrate Zoology, Moscow State University, Moscow 119899 Russia.; 18 Institute of Wildlife Biology and Game Management, University of Natural Resources and Life Science, A-1180 Vienna, Austria.; 19 Behavioural Ecology Group, Department of Animal Sciences, Wageningen University, Wageningen 6708 PB, The Netherlands.; 20 Department of Zoology and Laboratory of Ornithology, Faculty of Science, Palacký University, Olomouc 77147, Czech Republic.; 21 Department of Zoology, Institute of Ecology and Earth Sciences, University of Tartu, Vanemuise 46, Tartu 51014, Estonia.; 22 Institute of Biodiversity, Animal Health and Comparative Medicine, University of Glasgow, Glasgow G12 8QQ, UK.; 23 Department of Life Sciences, Institute of Marine Research IMAR/CMA, University of Coimbra, Coimbra, Portugal.; 24 Department of Biology, University of Oulu, P.O. Box 3000, 90014 Oulu, Finland.; 25 Evolutionary Ecology Lab, Institute of Ecology and Evolution, University of Bern, Bern 3012, Switzerland.; 26 Centre for Ecological and Evolutionary Synthesis (CEES), Department of Biosciences, University of Oslo, P.O. Box 1066, Blindern, 0316 Oslo, Norway.; 27 Centre for Ecological and Evolutionary Studies (CEES), Univ. of Groningen, PO Box 11103, NL-9700 CC Groningen, The Netherlands.; 28 Behavioural Ecology Group, Department of Systematic Zoology and Ecology, Eötvös Loránd University, Budapest H-1117, Hungary.; 29 Institute of Evolutionary Biology and Environmental Studies, University of Zurich, Winterthurerstrasse 190, CH-8057 Zurich, Switzerland.; 30 Graduate School of Environmental Science, Hokkaido University, N10 W5 Sapporo, Hokkaido 060-0810, Japan.

## Abstract

For over 50 years, the great tit (*Parus major*) has been a model species for research in evolutionary, ecological and behavioural research; in particular, learning and cognition have been intensively studied. Here, to provide further insight into the molecular mechanisms behind these important traits, we *de novo* assemble a great tit reference genome and whole-genome re-sequence another 29 individuals from across Europe. We show an overrepresentation of genes related to neuronal functions, learning and cognition in regions under positive selection, as well as increased CpG methylation in these regions. In addition, great tit neuronal non-CpG methylation patterns are very similar to those observed in mammals, suggesting a universal role in neuronal epigenetic regulation which can affect learning-, memory- and experience-induced plasticity. The high-quality great tit genome assembly will play an instrumental role in furthering the integration of ecological, evolutionary, behavioural and genomic approaches in this model species.

## Abstract (web summary)

The great tit (*Parus major*) is known for its complex social-cognitive behaviour. Here, the authors sequence genomes of the great tit and show genes related to learning and cognition in regions under positive selection, as well as neuronal non-CpG methylation patterns similar to those observed in mammals.

## Graphical abstract

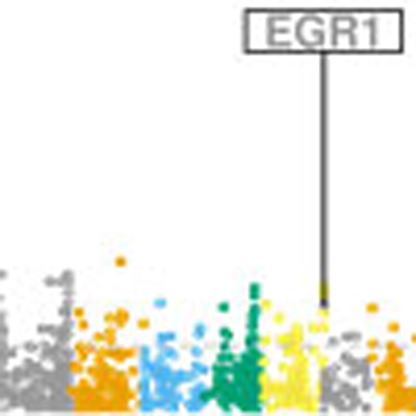

Theory predicts that the ability to perceive, assess and learn from others should have fitness benefits under a wide range of conditions^1^. However, we know little about whether, or how, natural selection acts on cognitive traits related to social living in any species^2^. Great tits learn socially in the wild^3^, solve complex learning tasks^4^ and there is evidence that cognitive abilities may have important fitness implications^5^. A literature survey of records of technical (for example, tool use) and opportunistic (for example, exploring novel food items) innovation spanning 803 bird species from 76 families shows that great tits are among the top avian species in terms of overall number and diversity of foraging innovations^6^. This suggests rapid evolution in the great tit lineage of learning and cognition-associated traits compared with many other birds, making great tits an excellent model for studying complex social-cognitive behaviour.

In addition to studies of cognitive and learning abilities of great tits^3^,^5^, great tit research has contributed significantly to our general understanding of life history evolution^7^, the effects of climate change on natural populations^8^,^9^, the allocation of resources to breeding^10^ including trade-offs between reproduction and immunity^11^, the extent and consequences of individual variation in rates of ageing^12^, inbreeding and inbreeding depression^13^,^14^, host–parasite coevolution^15^, territorial and foraging behaviour^16^,^17^ and the impact of variation in personality traits on other life history characters^18^. This considerable contribution of studies of great tits to our understanding of basic ecological and evolutionary processes is largely due to work on numerous long-term study systems throughout Europe.

To explore the molecular basis for learning and cognition, we developed a complete set of *de novo* molecular genomic tools to ascertain evidence of natural selection on genetic and epigenetic variation in great tits. Here, we test the hypothesis that learning and cognition have been important targets of selection in great tit evolution by exploring footprints of selection in the great tit genome. We find an overrepresentation of genes related to neuronal functions, learning and cognition in regions under positive selection, as well as increased CpG methylation in these regions. In addition, great tit neuronal non-CpG methylation patterns are very similar to those observed in mammals. The development of high-quality genomic resources for great tits provides tools that will allow the integration of genomics into ecological, evolutionary and behavioural research in wild populations in this important study system.

## Results

### Genome sequencing, assembly and annotation

We selected a male great tit (hereafter the reference bird) from a recent captive population (four generations in captivity) in the Netherlands for genome sequencing and *de novo* assembly (see Methods and Supplementary Information for a detailed description of genome assembly and annotation). We generated a total of 114 Gb of Illumina HiSeq sequence data; after gap-filling and removal of adaptor sequences, the assembly consisted of a total of 2,066 scaffolds with an N50 scaffold length of over 7.7 Mb and an N50 contig length of 133 kb. Taken together, the assembled contigs span 1.0 Gb. We were able to assign 98% of the assembled bases to a chromosomal location using the recently described high-density linkage map^19^ (Supplementary Data 1). The total number of chromosomes covered by the assembly is 29 with three additional linkage groups (Chr25LG1, Chr25LG2 and LGE22). The assignment of chromosomal locations to the majority of the assembly resulted in a very high quality reference genome and enabled detailed comparisons with other bird genomes. A comparison between the genomes of the great tit and zebra finch (*Taeniopygia guttata*)^20^ (see Supplementary Fig. 1) showed only intra-chromosomal inversions, and not a single inter-chromosomal rearrangement, confirming high synteny in birds. For the genome annotation step, we combined RNA sequencing data from eight different tissues of the reference bird with gene models from chicken (*Gallus gallus*)^21^ and zebra finch^20^. The final annotation resulted in a total of 21,057 transcripts for 13,036 high-confidence gene predictions.

### Population genomics

**Figure 1:**
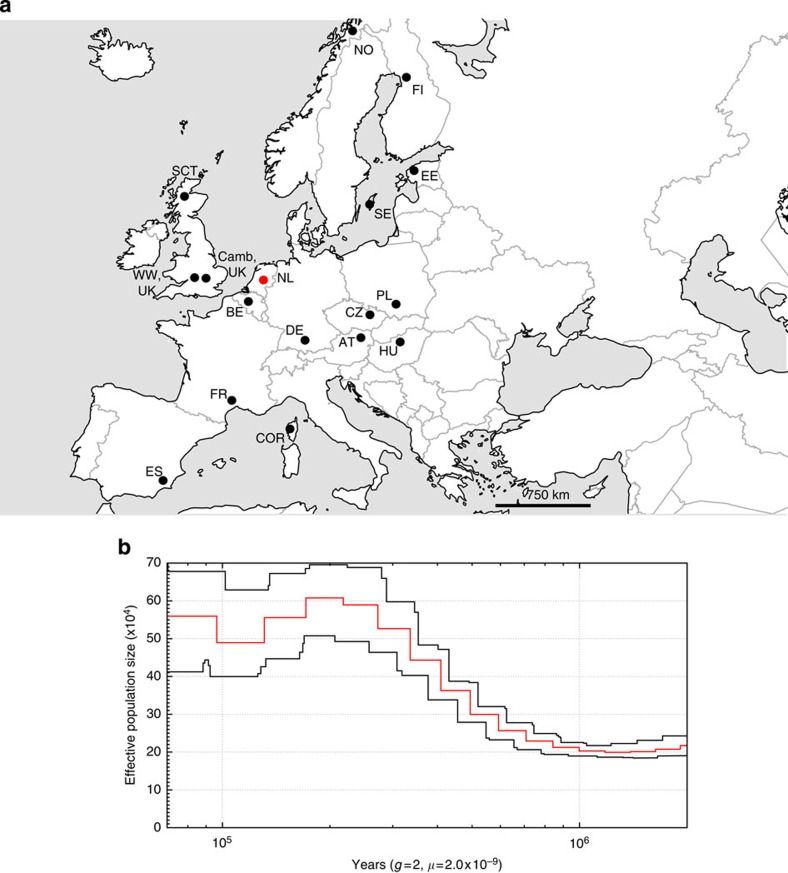
The 29 re-sequenced great tits and their demographic history. (**a**) Map of the sampling locations of the 29 re-sequenced great tits (black) and reference individual (red). (**b**) Pairwise sequential Markovian coalescent analysis (PSMC) of the reference genome. The red line represents the average and the black lines indicate the confidence interval as determined by bootstrapping (100 × ).

**Figure 2:**
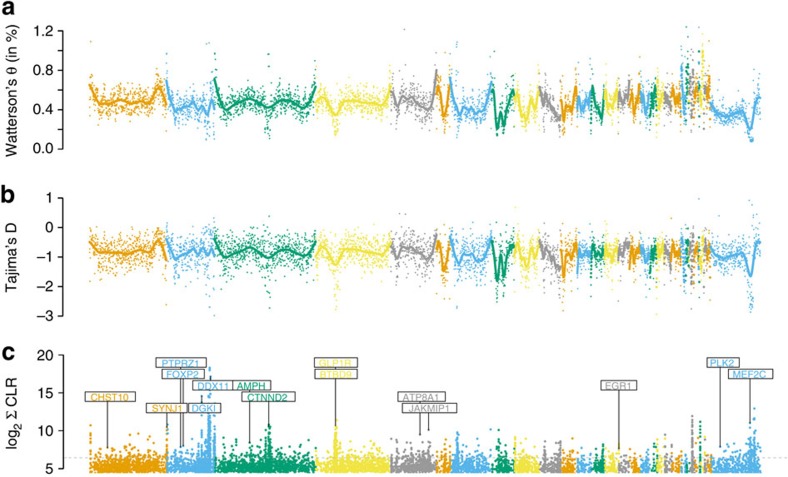
Genome-wide test statistics obtained from 29 re-sequenced great tits. (**a**) Genome-wide distribution of Watterson's Θ and (**b**) Tajima's D measured in sliding window sizes of 50 kB and step size of 10 kB, as well as (**c**) CLR (composite likelihood score, measured as the sum of neighbouring sweep targets, see Methods) from the sweep analysis with labelled cognition-related genes (see Fig. 3 and Supplementary Data 5a) that were among the top 3% of gene-associated sweep targets (indicated by the dashed line). Chromosomes are separated by colour in ascending order according to their chromosome number. The Z chromosome is the furthest right. The solid lines in the upper two panels denote smoothing splines.

To obtain further insight into the evolutionary genetics of the great tit, an additional 29 wild individuals from across Europe were re-sequenced at an average depth of 10 × (Supplementary Data 2, Fig. 1a). We found very little differentiation among populations (*F*_ST_<0.02; Supplementary Data 3) which is in line with previous phylogenetic analyses of the spatial genetic structure of great tits in Europe using mtDNA markers^22^. This suggests a largely panmictic European great tit population^22^. Pairwise sequential Markovian coalescent analysis (PSMC)^23^ of the reference individual suggested a large effective population size that increased from an already large ∼2 × 10^5^ individuals 1 Myr ago to ∼5.7 × 10^5^ individuals 70 Kyr ago (Fig. 1b). There was evidence that the expansion was interrupted by a very mild decline in population size beginning ∼110 Kyr ago, coinciding with the start of the last glacial period in Europe, followed by a quick recovery. Consistent with expectation for a population that has undergone a recent increase, there is a genome-wide excess of low-frequency variation, as indicated by negative Tajima's *D* values (Fig. 2b).

### Selective sweeps

**Figure 3:**
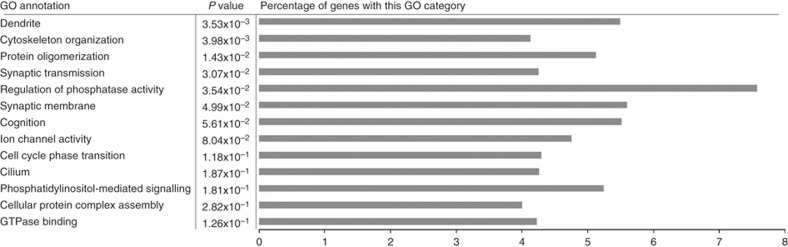
Gene ontology (GO) enrichment analysis of sweep area genes by using human gene ontologies. The GO enrichment analysis detected 13 functional groups of GO terms across all sweep areas (first column). *P* value denotes the corrected *P* value by using Bonferroni step-down method.

We found that several regions with reduced diversity coincide with regions of extremely negative Tajima's *D* (Fig. 2a,b), indicative of recent selective sweeps. To investigate the role of recent positive selection in greater detail, we conducted a genome-wide scan for selective sweeps and identified 813 genomic regions (Fig. 2, Supplementary Data 4) compromising roughly 0.2% of the genome. We extracted 460 genes that had human orthologues from these regions and assessed their functional importance by performing a gene ontology (GO) enrichment analysis. Thirteen different GO categories were overrepresented in these selected regions relative to the rest of the genome (Fig. 3, Supplementary Data 5a). Among these categories were dendrite, cognition and several other categories related to neuronal function. Therefore genes related to neuronal function are likely to have been targets of recent positive selection.

### Learning and cognition

One of the striking genes presented in the learning/cognition category was early growth response protein 1 (*EGR1*), whose gene expression has been particularly well studied within vocal communication and social contexts in Passerines^24^,^25^. *EGR1* is one of the immediate early genes, which are known to be important in learning and memory^26^. In zebra finches, its expression has been shown to be correlated with song learning and practice and is social context dependent^25^,^27^. To further investigate the role of *EGR1* in great tit evolution, we obtained additional EGR1 sequences from 45 bird species (Supplementary Data 6). We found that rapid evolution of *EGR1* is specific to the tit lineage (as signified by an elevated ratio of non-synonymous to synonymous substitution rate (dN/dS)) and has occurred after the split with the nearest sequenced relative, the ground tit and that this is not a feature related to the captive nature of the reference bird (Supplementary Fig. 2). These results are consistent with *EGR1* being subject to frequent, recurrent positive selection. Another relevant gene from this category was the forkhead box protein P2 (*FOXP2*), a well-studied gene that affects speech and language development in humans^28^ and linked to song learning in birds^29^,^30^. The combination of these song-related genes and the other neuronal genes within sweep areas points to a role of these genes in the evolution of song learning and memory in great tits.

### Rates of molecular evolution

To examine whether the identified GO categories in general play an important role in great tit evolution, we extracted all great tit genes that are associated with the enriched GO terms of the sweep regions (Fig. 3, Supplementary Data 5a) and conducted evolutionary rate (dN/dS) analyses by using orthologous genes from chicken and zebra finch. We found that median dN/dS for genes associated with the identified GO terms is reduced compared with all other GO-annotated genes (0.066 versus 0.087, *P*=1.4 × 10^−22^, Mann–Whitney *U*-test), and yet these genes have significantly more targets of probable positive selection based on a site test of positive selection (*P*=1.84 × 10^−3^, *χ*^2^ test; see Methods for details). Moreover, on a genome-wide scale, ∼1% of the genes in the great tit genome show evidence for positive selection based on their long-term evolutionary rates. These genes are generally enriched for neuronal traits, such as cerebellar Purkinje cell layer formation and axon extension (Supplementary Data 5b). Taken together, it becomes apparent that selection on traits for brain function has played a major role in the evolution of great tits.

### The great tit methylome

**Figure 4:**
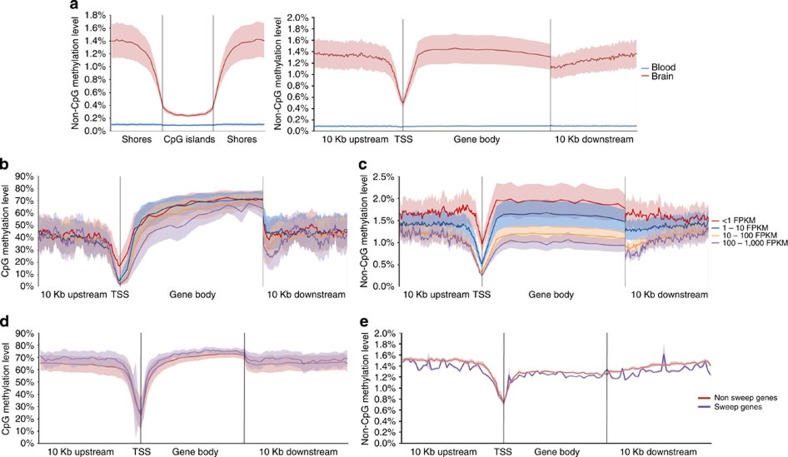
DNA methylation patterns across genomic features. (**a**) Non-CpG methylation patterns associated with CpG islands and gene bodies. (**b**) Neuronal CpG methylation in gene bodies and at TSS is negatively correlated with expression (Spearman's rank correlation, Spearman's rho <−0.23, *P*<1.0 × 10^−95^ for all comparisons), presented as fragments per kilobase of transcript per million fragments mapped (FPKM). (**c**) Neuronal non-CpG methylation at TSS, gene bodies and adjacent upstream and downstream regions is negatively correlated with expression (Spearman's rho <−0.23, *P*<1.0 × 10^−95^ for all comparisons). (**d**) Increased neuronal CpG methylation at sweep gene bodies (Linear Mixed Effect Model, LMM; 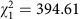, *P*=9.2 × 10^−88^) and adjacent upstream (LMM; 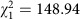, *P*=3.0 × 10^−34^) and downstream regions (LMM; 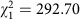, *P*=1.8 × 10^−65^). (**e**) Decreased neuronal non-CpG methylation in sweep gene bodies (LMM; 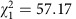, *P*=4.0 × 10^−14^) and adjacent upstream (LMM; 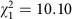, *P*=0.001) and downstream regions (LMM; 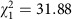, *P*=1.64 × 10^−8^). Shaded areas denote variances.

Epigenetic control of gene expression is increasingly recognized as playing a major role in many different cellular processes affecting a large variety of traits^31^, with DNA methylation of cytosines being the most widely studied epigenetic mark. Great tit DNA methylation patterns were investigated by performing whole-genome bisulfite sequencing in whole brain and blood tissue of the reference bird. A total of 10.2 million CpG sites, representing 66.7% of all CpG sites, could be called in both tissues. The observed genome-wide methylation patterns in both tissues, including reduced methylation within CpG islands and at transcription start sites (TSS), are consistent with previous findings in human and mouse cells^32^,^33^(Supplementary Fig. 3). We also observed low, but significant non-CpG methylation in the brain tissue that was not observed in the blood (Fig. 4a). The neuronal non-CpG methylation patterns seen at 167.4 million sites (42% of all non-CpG sites) display similar genome-wide patterns to those seen for CpG methylation, including reduced methylation within CpG islands and at TSS, albeit at a much lower level. Similar non-CpG methylation has recently been found in embryonic stem cells and oocytes of many mammalian species^34^,^35^, as well as in the neuronal tissue of humans and mice^33^,^35^.

Both CpG and non-CpG methylation has been shown to negatively correlate with gene expression at TSS and in gene bodies in mouse neurons^33^, while hypomethylation of both CpG and non-CpG sites in gene bodies has been reported in highly expressed genes of human neurons^35^. Consistent with these results, both CpG and non-CpG methylation were negatively correlated with gene expression within gene bodies and at TSS in the great tit brain (Spearman's rho < −0.23, *P*<1.0 × 10^−95^ for all comparisons, Fig. 4b,c). In addition, the negative correlation between non-CpG methylation and expression was observed in the regions directly upstream and downstream of gene bodies (Spearman's rho <−0.22, *P*<0.0001 for all comparisons, Fig. 4c). Therefore, our findings now extend a potential functional role of non-CpG methylation in neuronal tissue to Aves, suggesting evolutionary conservation of this epigenetic regulation in brains.

### Methylation is correlated with rates of molecular evolution

To investigate the potential adaptive and evolutionary role of DNA methylation, we first assessed the methylation patterns of selective sweep genes in the brain. We observed higher CpG methylation at sweep gene bodies (Linear Mixed effect Model, LMM; 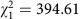, *P*=9.2 × 10^−88^) and in regions upstream (LMM; 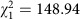, *P*=3.0 × 10^−34^) and downstream (LMM; 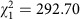, *P*=1.8 × 10^−65^) of gene bodies compared with the same regions in genes outside sweep regions (Fig. 4d). In addition, lower non-CpG methylation in sweep gene bodies (LMM; 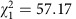, *P*=4.0 × 10^−14^) and in regions upstream (LMM; 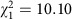, *P*=0.001) and downstream (LMM; 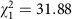, *P*=1.64 × 10^−8^) of gene bodies was observed compared with the same regions in genes outside sweep regions (Fig. 4e). These patterns are not due to systematic differences in expression levels between sweep and non-sweep genes because the observed CpG and non-CpG methylation differences are in opposing directions. In addition, although the overall expression profiles reveal a higher proportion of non-sweep genes with no or low expression (Supplementary Fig. 4e), differences seen between CpG and non-CpG methylation in sweep gene regions were also observed when comparing genes with similar expression levels (Supplementary Fig. 4a–d). While previous studies in chickens and humans have also shown altered CpG methylation in regions under selective pressure^36^, it is unclear what causes the observed higher CpG and lower non-CpG methylation in great tit sweep regions. In addition, lowly methylated genes in the brain were found to evolve significantly slower in comparison with highly methylated genes as shown by differences in the rate of nonsynonymous mutations between the two groups of genes (*P*<0.001 for all pairwise comparisons, *U*-test, Supplementary Fig. 5). This pattern was observed for both CpG and non-CpG methylation at TSS and within gene bodies. Overall these results not only show, for the first time, conserved neuronal non-CpG methylation patterns between birds and mammals, but also extend them by showing that methylation is correlated with rates of molecular evolution, thereby suggesting an important role for DNA methylation in evolution.

## Discussion

We have generated a high quality *de novo* assembled and annotated genome for the great tit, a model organism in ecology and evolutionary biology. Our study adds to the growing number of representative genomes sequenced across the bird family tree^37^,^38^. Further, unlike these recent genome studies, we assembled the great tit genome into chromosomes, which was useful for determining chromosomal regions of selective sweeps and larger-scale synteny conservation across species. The great tit genome assembly represents a powerful tool, especially when combined with the extensive availability of individual phenotypes of known birds for which DNA is available via routine blood sampling. Using sequence analysis of birds from a wide range of European populations, we identified selective sweep areas enriched with genes related to learning and cognition. Using whole-genome methylation data, we not only revealed conserved non-CpG methylation patterns between birds and mammals, but also extended these observations by showing that methylation is correlated with rates of molecular evolution, thereby suggesting an important role for DNA methylation in evolution. Our *de novo* assembled genome will help us to reveal the genetic basis of phenotypic evolution, which is essential for understanding how wild species have adapted to our changing planet.

## Methods

### Genome sequence assembly and annotation

A blood sample from a male *Parus major* was obtained for whole-genome sequencing and stored in queens buffer. This reference bird originated from a captive population that was derived from wild-caught birds from the Netherlands four generations ago and has since been artificially selected for avian personality^39^. In the great tit genome assembling, we relied on the creation of de Bruijn graphical structures, a directed graph that evolves from defined sequence length (kmer) progression (see Supplementary Methods for a detailed description of genome assembly and annotation). In brief, our genome assembling involved four principal steps that progressed from sequence quality revisions, to forming contigs from these sequence reads, to connecting contigs into scaffolds using paired-end sequence of large fragments (jumping libraries) and finally gap filling. In this study, the total input genome coverage of Illumina HiSeq sequences was ∼95 × (fragments, 3 and 8 kb spanning inserts) based on a genome size estimate of 1.2 Gb. The combined sequence reads were assembled using the ALLPATHS software^40^. This draft assembly was referred to as Parus_major 1.0.1. This version has been gap filled and cleaned of contaminating contigs. The assembly is made up of a total of 2,066 scaffolds with an N50 scaffold length of over 7.7 Mb (N50 contig length was 133 kb). The total assembled contigs spans 1.0 Gb, and has an assembled coverage of 40 × using fragment reads aligned to the assembly. The final assembly (Pmajor1.04) has been deposited at DDBJ/EMBL/GenBank under the accession JRXK00000000. The version described in this paper is version JRXK01000000. The genome is also publicly available in https://genomes.bioinf.nioo.knaw.nl/.

### Assembly chromosome builds

Assembled scaffolds were assigned to specific chromosomes using two *Parus major* linkage maps^19^ constructed from different populations; one at Wytham Woods, Oxford (UK) and the other at de Hoge Veluwe (Netherlands), which is the population that the reference bird descended from. Flanking sequences of single-nucleotide polymorphisms (SNPs) positioned on the linkage map were aligned against the assembled scaffolds using BLAT. Scaffolds that contained multiple SNP markers were then oriented and positioned on the basis of the positions of the SNP markers on the linkage map. The order of the SNP markers on the scaffolds and the linkage map were in good agreement (Supplementary Fig. 6). Regardless of whether the Netherlands or UK map was used, the Spearman rank correlation coefficient between the linkage map marker order and the assembly marker order was 0.99 or greater for nearly all the chromosomes. Exceptions were only on microchromosomes with just a few markers, for example, linkage group 25LG22. If anything, correlation coefficients were slightly higher when the UK map was used, despite the genome assembly being performed on a Netherlands bird. This indicates that the assembly is equally applicable to other great tit populations as it is to the ‘source' population. Most discrepancies between the orders on the sequence and linkage map were caused by the lower resolution of the linkage map involving SNPs that were less than 1 cM apart. Two scaffolds (22 and 28) appeared to be chimeric and were manually split between contigs Contig22.251-Contig22.250 and contigs Contig28.53-Contig28.51, respectively. Because of a lack of any genetic marker on Contig28.52, this contig was not assigned to a specific chromosome.

Small scaffolds that were assigned to a chromosome based on only a single marker were oriented based on the zebra finch-great tit comparative map, taking into account the orientation of the flanking scaffolds assigned to that chromosome. Next, we used MUMMER to align all unassigned scaffolds against the zebra finch genome^20^ and larger scaffolds that unambiguously mapped to a specific region of the zebra finch genome at a location between assigned scaffolds were assigned to that location in the genome. The orientation of these scaffolds was chosen in relation to the adjacent mapped scaffolds, thus minimizing the number of rearrangements. In total, 300 scaffolds could be assigned to a chromosomal location based on the linkage map^19^ totalling 975,330,736 bp and 124 scaffolds were assigned to a chromosomal location based on the alignment with the zebra finch genome, totalling 23,489,268 bp (Supplementary Data 1). Although the final genome assembly therefore is not completely *de novo*, these contigs only represent 2.4% of the assembled sequence and 97.6% of the assembly represents a truly *de novo* genome assembly.

### RNA sequencing and assembly

RNA was extracted from eight tissues (bone marrow, homogenized half of the brain, breast filet, higher intestine, kidney, liver, lung and testis) from the reference bird and was then used to prepare tissue-specific tagged Illumina sequencing libraries. The tagged libraries were pooled and sequenced using five lanes on one flowcell of Illumina HiSeq 2000 (same run on the machine). This resulted in 100 bp paired-end unstranded RNA sequencing data. The number of reads per tissue ranged from 98 to 229 million with a total number of 1.25 billion paired-end reads (Supplementary Data 7). The sequence reads were checked for quality with FastQC (http://www.bioinformatics.babraham.ac.uk/projects/fastqc/) and low-quality sequences were trimmed with Fastq-Mcf^41^ resulting in a final number of 1,096,140,415 paired-end reads that were used for the annotation. The RNA sequencing data per individual tissue has been submitted to Genbank (GT_BoneMarrow SRS863935, GT_Brain SRS866013, GT_BreastFilet SRS86603, GT_HigherIntestine SRS866033, GT_Liver SRS866035, GT_Kidney SRS866036, GT_Lung SRS866044, GT_Testis SRS866048).

The combined 1 billion reads from all eight tissues were simultaneously *de novo* assembled using the Trinity software package^42^ v. r2013-02-25. Because of the high depth of the RNA sequencing data, we first normalized the data. Following the normalization, the Trinity assembly was subsequently run using the default settings. We obtained a total of 101,289 assembled transcripts ranging in size from 201 to 16,061 bp, with an average size of 1,335 bp. We also did a reference-based RNA assembling by using the normalized RNA sequencing data in Tophat version v2.0.10 (ref. 43; Bowtie v2.1.0 (ref. 44)).

### Genome annotation

For the genome annotation, both PASA v. 2-r20130605 (ref. 45) and MAKER v. 2.31.5 (ref. 46) pipelines were used (Supplementary Fig. 7). First PASA (program to assemble spliced alignments) was used for the identification of spliced transcripts and the grouping of the identified transcripts belonging to the same gene. PASA was run using the assembled Trinity transcripts and the Cufflinks gtf output file from Tophat/Bowtie as input. Alignment within PASA was done using gmap. The PASA analysis resulted in a total of 74,229 different assembled transcripts with an average size of 1,564 bp. A comparison (using blast) with the annotated transcriptomes of chicken^21^, zebra finch^20^, flycatcher^47^ and ground tit^48^ (derived from Ensembl release 75 or NCBI refseq release 66) indicated that these transcripts represented 13,626 different genes in these other birds.

In the MAKER pipeline, the output of PASA was used as EST evidence. From the *ab initio* predictors, AUGUSTUS version 3.0.2 (ref. 49) was applied by using the chicken gene model. The same RNA-seq *de novo* assembly as used in PASA was included in MAKER. In addition to *de novo* assembly, reference-based RNA-seq assembly was used. Last, protein evidence from zebra finch (version 3.2.4.75) and chicken (version 4.75) obtained from Ensembl version 75 was aligned to the great tit genome. By combining these two pipelines, we obtained 21,057 transcripts for 13,036 great tit genes. See additional information about functional annotation and repeat/RNA masking in Supplementary Information and Supplementary Data 8.

### Resequencing and SNP calling

We analysed 29 wild great tit individuals (Supplementary Data 2) covering a wide range of the species distribution (Fig. 1); 10 individuals were from the Wytham population in Oxford (UK), and the remaining 19 birds were sampled from 15 European populations. Each bird was sequenced to ≈10 × coverage. Paired-end sequencing libraries of each sample were built with an insert size of 300 bp and sequencing was performed on a HiSeq 2000 platform with a read length of 100 bp. The raw reads were trimmed and filtered with Sickle (https://github.com/najoshi/sickle) using default parameters and a length restriction of 75. We then used the Burrows–Wheeler Aligner^50^ to map the filtered raw reads onto the assembled great tit reference genome. Subsequently, we removed duplicates and conducted local realignments following the best practices of the GATK pipeline^51^. We used ANGSD^52^ to call SNPs based on the genotype likelihoods estimated by the GATK model from the mapped reads^51^; this approach has been shown to produce more accurate estimates of the Site Frequency Spectrum (SFS) than other widely used SNP-calling pipelines when sequencing coverage is low (<10 × )^53^, which is critical for our analysis because our average coverage is 10 × . However, for comparison, we also called SNPs using three additional approaches: (1) GATK using data from each individual separately (the ‘Single' approach); (2) GATK using data from all individuals simultaneously (the ‘Multi' approach); (3) Platypus, which calls SNPs directly from the Burrows–Wheeler Aligner alignments, independent of the GATK pipeline^54^. We also tested the reliability of the SNP data, see details in Supplementary Information and Supplementary Data 9 and 10 and Supplementary Fig. 9.

### Population differentiation and demography

We found very little differentiation among populations (*F*_ST_<0.015; Supplementary Data 3). The most differentiated population pairs were Spain–UK (ES versus WW, UK; Fig. 1) and France–Spain (FR–ES; Fig. 1) with *F*_ST_=0.012.

We analysed the demographic history with pairwise sequential Markovian coalescent analysis (PSMC^23^). PSMC estimates rates of coalescent events across a single genome and uses these to infer *N*_e_ (the effective population size) in the past^23^. The model relies significantly on confidently called polymorphic sites and requires that both alleles are called. Therefore we conducted this analysis with variants called on the high coverage reference genome sequence. We set the mutation rate to 2.0 × 10^−9^ per year per site and the generation time to 2 years.

### Genome-wide diversity and Tajima's D

We obtained genome-wide sliding window estimates (step size 10 kb, window size 50 kb) of Watterson's Θ (Fig. 2) and Tajima's *D* (Fig. 2) along each chromosome based on the SNP calls from ANGSD. For most macrochromosomes, diversity is increased towards the chromosome ends, and there are remarkable local drops of diversity on chromosomes 3, 6 and Z (Fig. 2; more details about low diversity in chromosome Z in Supplementary Information and Supplementary Fig. 10). By calculating Watterson's Θ for synonymous sites in protein-coding genes we found a clear negative correlation between chromosome length and diversity levels (Supplementary Fig. 8); we observed the same pattern when diversity was estimated using all sites (Supplementary Fig. 8).

### Selective sweep detection

We scanned the genome for target regions of positive selection using Sweepfinder^55^ which uses local deviations of the site frequency spectrum (SFS) compared with a standard neutral model or chromosome/genome-wide reference to infer the action of positive selection. We used SNP calls from the ANGSD pipeline to construct SFS. ANGSD assumes that each variable position is biallelic and determines allele frequencies based on the genotype likelihoods^56^. Whole-genome alignments constructed with MAUVE^57^ for zebra finch^20^, flycatcher^47^, ground tit^48^ and chicken^21^ were used to infer the ancestral state based on maximum parsimony. Sites for which the ancestral state could not be reconstructed with confidence were included in the analysis as folded sites (unpolarized). The chromosome-wide SFS was used as reference for each chromosome as recommended by Sweepfinder, which helps to reduce false positives caused by past demographic changes. We used a dense grid size of 100 bp and extracted sweep targets that had a composite likelihood score (CLR) within the top 1%. Neighbouring sweep targets were merged to sweep regions, with the total CLR score of a sweep region being the sum of the CLR score of each sweep target. The top 3% of genes that overlapped with or were near (within 5 kB flanking the sweep region) sweep regions larger than 300 bp were extracted (Supplementary Data 4). Targets for positive selection included the low diversity region of chromosome 3, 6 and Z. Further details of the Sweepfinder pipeline can be found in the Supplementary Materials and Methods.

### Multiple alignment construction for substitution rate analyses

Natural selection affects the composition of genomes and we were interested in estimating gene-specific rates of molecular evolution (dN/dS) and finding evidence for the action of positive selection in protein coding genes. Since alignment quality is crucial for reliably conducting tests of positive selection using substitution rate analyses, we chose a very conservative approach. We used homologous genes from zebra finch and chicken, the two best annotated bird genomes to date. We only analysed genes with a homologue in both, and used MUSCLE^58^ along with ZORRO^59^ to exclude positions of low alignment certainty. To obtain the corresponding multiple DNA codon alignments, protein alignments along with the unaligned DNA sequences were prepared with PAL2NAL^60^. Altogether, we constructed ≈11,107 triplet alignments. Substitution rates were calculated using PAML^61^ to obtain the nonsynonymous to synonymous substitution rate ratio (dN/dS=*ω*). *ω* values <1, =1 and >1 indicate purifying selection, neutral evolution and diversifying (positive) selection, respectively. Rates of molecular evolution (dN/dS) for each gene were obtained from the one-ratio model M0 from PAML that assumes a constant *ω* for the whole gene phylogeny. In addition, a site test was used to detect positive selection. Specifically, we compared the likelihoods calculated using model M8, which assumes a proportion of sites to evolve under positive selection, and model M7, which does not assume a site class with *ω* exceeding one. Positive selection was inferred when the model M7 and M8 were significantly different as assessed by a likelihood-ratio test that assumes that 2ΔlnL (twice the log likelihood difference) is *χ*^2^ distributed with two degrees of freedom. The *P* values were adjusted for multiple testing (Benjamini and Hochberg) with a false discovery rate of 0.2; *χ*^2^ test results for an enrichment of positively selected genes were qualitatively similar for FDRs=0.1, 0.3, 0.4 and 0.5 (*P*=2.17 × 10^−5^, 3.97 × 10^−3^, 2.58 × 10^−3^ and 2.17 × 10^−3^, respectively).

### GO enrichment analyses

Human orthologues were obtained for the great tit genes by using a combination of Ensembl and Uniprot databases. In the sweep areas, orthologues were found for 460 genes. Functional relatedness of GO terms was done using the Cytoscape plugin ClueGo 2.1.4 (ref. 62). ClueGo constructs and compares networks of functionally related GO terms with kappa statistics. A two-sided hypergeometric test (enrichment/depletion) was applied with GO term fusion, network specificity was set to ‘medium' and false discovery correction was carried out using the Bonferroni step-down method. We used both human (08.10.2015) and chicken gene ontologies (08.10.2015) for comparison. With human gene ontologies, we detected 13 functional groups of GO terms across all sweep areas (Supplementary Data 5a). These groups were largely involved in functions concerning dendrite, cytoskeleton organization, protein oligomerization, synaptic transmission, regulation of phosphatase activity, synaptic membrane and cognition. We also did a GO enrichment analysis for the positively selected genes, as defined by the PAML-based analysis, in the same way as for the sweep genes, and obtained 12 functional GO groups (Supplementary Data 5b). When using the chicken orthologues for both sweep and positively selected genes, the results were comparable but with lower significance levels (Supplementary Data 5c,d) because the chicken genes were not as well GO annotated as the human ones.

To further confirm our GO enrichment results, we randomly selected 50 sets of genes from outside the sweep area, each containing ∼460 genes (the same number as in our selective sweep set), and analysed them using the same Cluego settings. We found that the GO term groups significantly enriched in our selective sweep set appeared no more than three times except for phosphatidylinositol-mediated signalling GO group which appeared seven times. This additional analysis further supports the robustness of our results.

### Test of accelerated evolution in great tit *EGR1*

We used BLAST to obtain orthologous sequences of the *EGR1* gene from 45 additional bird species^37^,^38^ downloaded from Ensembl version 75 or from Gigascience (http://gigadb.org/dataset/101000, Supplementary Fig. 2, detailed species list provided in Supplementary Data 6) to test whether there was additional evidence for the action of positive selection during the evolution of *EGR1* in birds. Pairwise dN/dS rates revealed that there is a substantial increase in dN/dS between great tit and ground tit relative to other pairwise values (Supplementary Fig. 2, upper panel). We also tested whether the increased fixations are unique to the captive reference bird by using variable positions from the 29 re-sequenced birds, the reference bird and the ground tit genome and found no evidence for this (Supplementary Fig. 2 lower panel).

### Brain gene expression

To compare expression levels and methylation in the brain, 200,793,186 trimmed paired-end reads from the brain were aligned against the assembled genome using Tophat version v2.0.10 (ref. 43; Bowtie v2.1.0 (ref. 44)) with the same settings as described above, except that multiple hits were prefiltered against the genome (-M option) and the reads were first aligned against the final annotation (-G option). The brain Tophat alignment was analysed with Cufflinks v2.2.0 using the same settings as above, except that the annotation was also included (-g option). Expression levels of brain genes were extracted from the Cufflinks output.

### Methylation analysis

Blood and brain DNA libraries were constructed according to the Epitect whole-genome bisulfite sequencing workflow (Illumina) with 18 PCR cycles. Whole-genome sequencing data were generated using the Illumina HiSeq 2,500 platform at Business Unit Bioscience, Wageningen UR. The number of paired-end reads (101 bp) were 358M and 292M for the brain and the blood, respectively. Raw sequencing reads were trimmed for quality (⩾20) and adaptor sequence using trim_galore v.0.1.4 (http://www.bioinformatics.babraham.ac.uk/projects/trim_galore/). The methylation data has been submitted to NCBI with accession numbers SRR2070790 and SRR2070791 for the brain and the blood, respectively. Trimmed sequences were aligned to the reference genome using BSseeker2 v2.0.6 (ref. 63) with Bowtie2 v.2.1.0 (ref. 44) in the local alignment mode. A total, 97.63% and 99.93% of the genome was covered to an average depth of 31.88 × and 33.04 × in brain and blood, respectively. Methylation levels for each site were determined using the BSseeker2 methylation call script. All the analyses were done using sites covered by a minimum of 10 reads in both the samples. Only genes found to be 1:1:1 orthologues with chicken and zebra finch were used for methylation analysis. Gene bodies (annotated gene boundaries excluding the 5′ 5% of genes) and TSS (300 bp upstream to 50 bp downstream of the annotated starting position of each gene) for which we had information from at least 50% of the potential methylation sites were used in the dN/dS and expression correlation analysis. Average methylation levels for TSS and gene bodies were calculated for each individual gene. The upper (highly methylated) and lower (lowly methylated) quartiles were compared for differences in their evolutionary rates (dN/dS) using a Mann–Whitney *U*-test. Correlations between the average methylation level of a given region (TSS or gene body) per gene with expression were performed using Spearman's rank correlation. A sliding window approach was used to infer differences in methylation levels between sweep and non-sweep gene regions. Non-sweep genes located on scaffolds were not included in the analysis, as these regions were not tested for sweeps. For this, genes were divided into different regions: the gene body (described above), 10 kb upstream and 10 kb downstream of the gene body and the TSS region (described above). Each gene body was subdivided into 40 bins, with the length of each bin therefore depending on the length of the gene. We calculated the mean methylation levels of these 40 bins with an overlap between neighbouring bins of 250 bp. Upstream and downstream regions were divided into bins of exactly 250 bp with an overlap of 125 bp between consecutive bins.

To compare TSS methylation between sweep and non-sweep genes, we conducted a *t*-test with equal variance assumed. Only TSS regions with a minimum of 10 covered sites were used for comparative analysis. To compare methylation levels between sweep and non-sweep genes for other gene regions, we conducted LMM analyses with methylation level as the dependent variable, sweep (yes or no) as a fixed factor and bin as a random factor. A likelihood-ratio test was conducted comparing the model with and without sweep as a fixed factor. All criteria were met for conducting parametric analyses.

## Additional information

**How to cite this article:** Laine, V. N. *et al*. Evolutionary signals of selection on cognition from the great tit genome and methylome. *Nat. Commun.* 7:10474 doi: 10.1038/ncomms10474 (2016).

## Supplementary Material

Supplementary Figures, Supplementary Methods and Supplementary References Supplementary Figures 1-10, Supplementary Methods and Supplementary References

Supplementary Data 1 Scaffolds

Supplementary Data 2 Sampling locations, sex, sample volumes and DNA concentrations of the 29 re-sequenced great tits

Supplementary Data 3 The pairwise FST values for five sample sites. EU samples exclude samples from Spain, Estonia and France individuals

Supplementary Data 4 Chromosomal locations of sweep areas, along with their summed composite likelihood ratio (CLR) score from the SweepFinder analysis.

Supplementary Data 5 Results of an enrichment test conducted for GO terms associated with the genes found from selective sweep and that were positively selected by using human orthologs (a and b) and chicken orthologs (c and d). From the random gene set test, the GO term groups appeared no more than three times except for phosphatidylinositol-mediated signalling GO group which appeared seven times.

Supplementary Data 6 Species from which EGR1 sequences were obtained.

Supplementary Data 7 The amount of RNA reads before and after filtering done by Fastq-Mcf.

Supplementary Data 8 Repeat content of the great tit genome identified by RepeatMasker

Supplementary Data 9 Chromosome wide diversity estimates (measured as Watterson's θ) from 29 birds (called with ANGSD) and from the reference bird ABEL (called with Platypus). The ratio of the two diversity estimates per chromosome is given along with the Spearman correlation coefficient of per chromosome diversity (measured in sliding windows of size 50 kb).

Supplementary Data 10 The number of SNPs for each chromosome from the four different SNP calling approaches, the recalibrated set and the consensus set (see Methods).
