# Supplementary material for: Evolutionary signals of selection on cognition from the great tit genome and methylome
Source: Nat Commun. 2016 Jan 25;7:10474. doi: 10.1038/ncomms10474 (PMC4737754; doi:10.1038/ncomms10474)
Supplement: Supplementary Figures, Supplementary Methods and Supplementary References — Supplementary Figures 1-10, Supplementary Methods and Supplementary References [file ncomms10474-s1.pdf]

Supplementary information

Supplementary figures

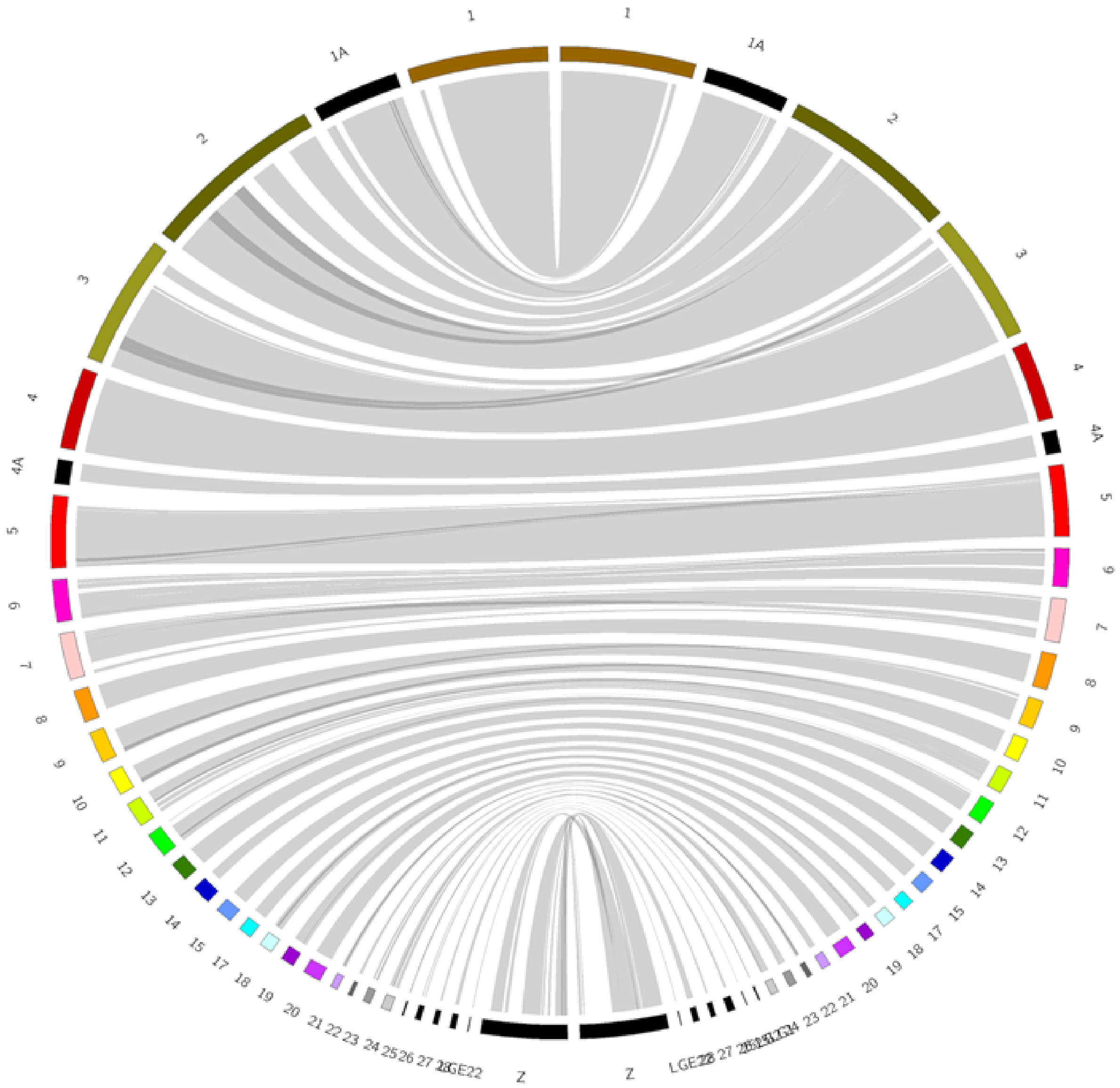

Supplementary Figure 1. Chromosomal synteny between zebra finch (left) and great tit (right) genome assemblies. Different colours represent different chromosomes. The genome comparison was done by using the software LASTZ[1].

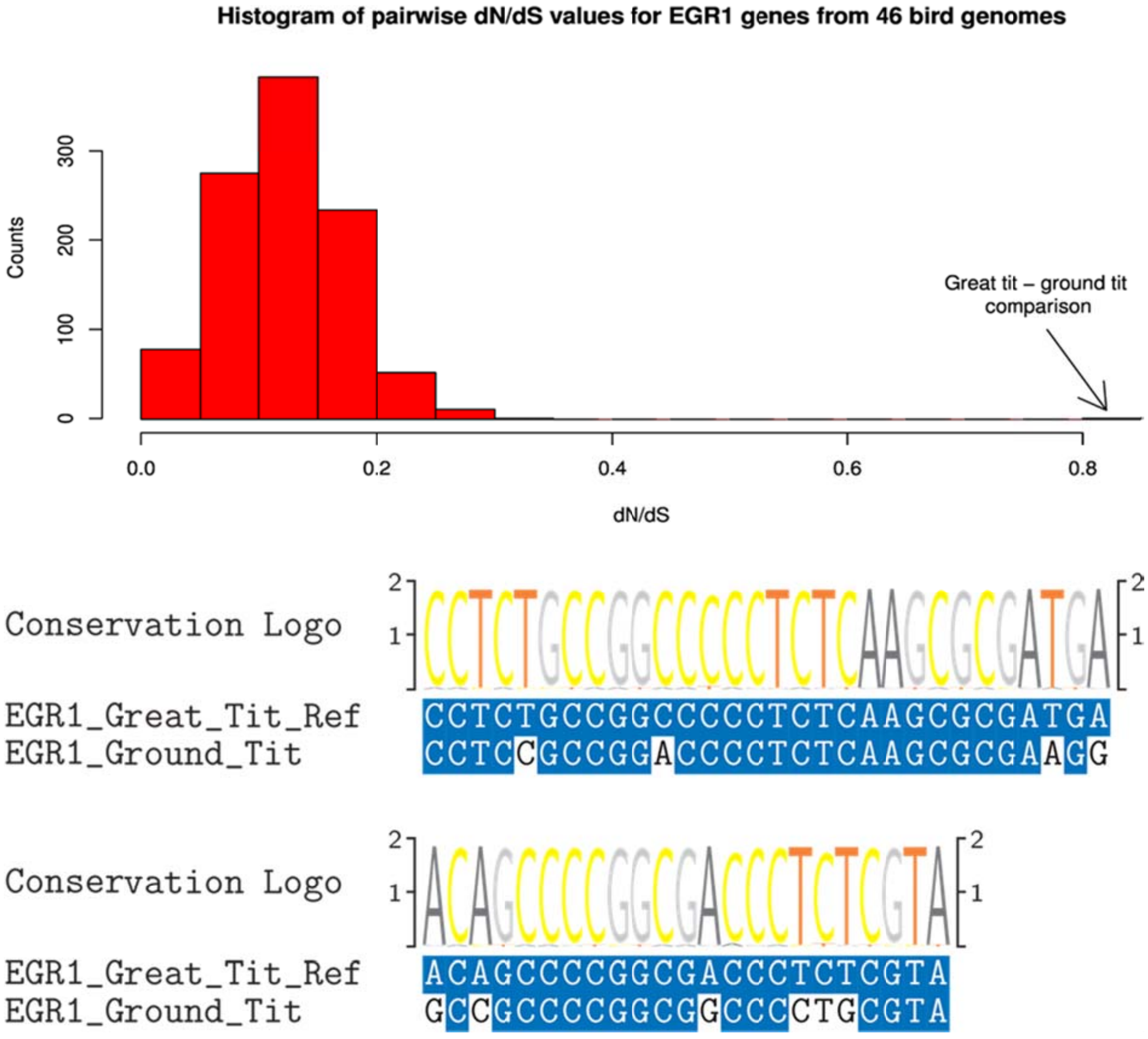

Supplementary Figure 2. Patterns of accelerated evolution of the EGR1 gene in the tit lineage. Upper Panel: Pairwise dn/ds rates between 46 bird genomes[2] (for a full list of species see Supplementary Data 6 of gene *EGR1*). Increased dN/dS value for the great tit-ground tit comparison is indicated with an arrow. Lower panel: Variable sites in the EGR1 coding region for the 29 birds, reference bird and ground tit EGR1 represented as a conservation sequence logo plot[3]. The sequence of the reference bird and ground tit is given below, and the major variant is highlighted in blue. Altogether 53 positions are variable, 10 are fixed differences between ground tit and great tit EGR1 and none is specific to the reference bird.

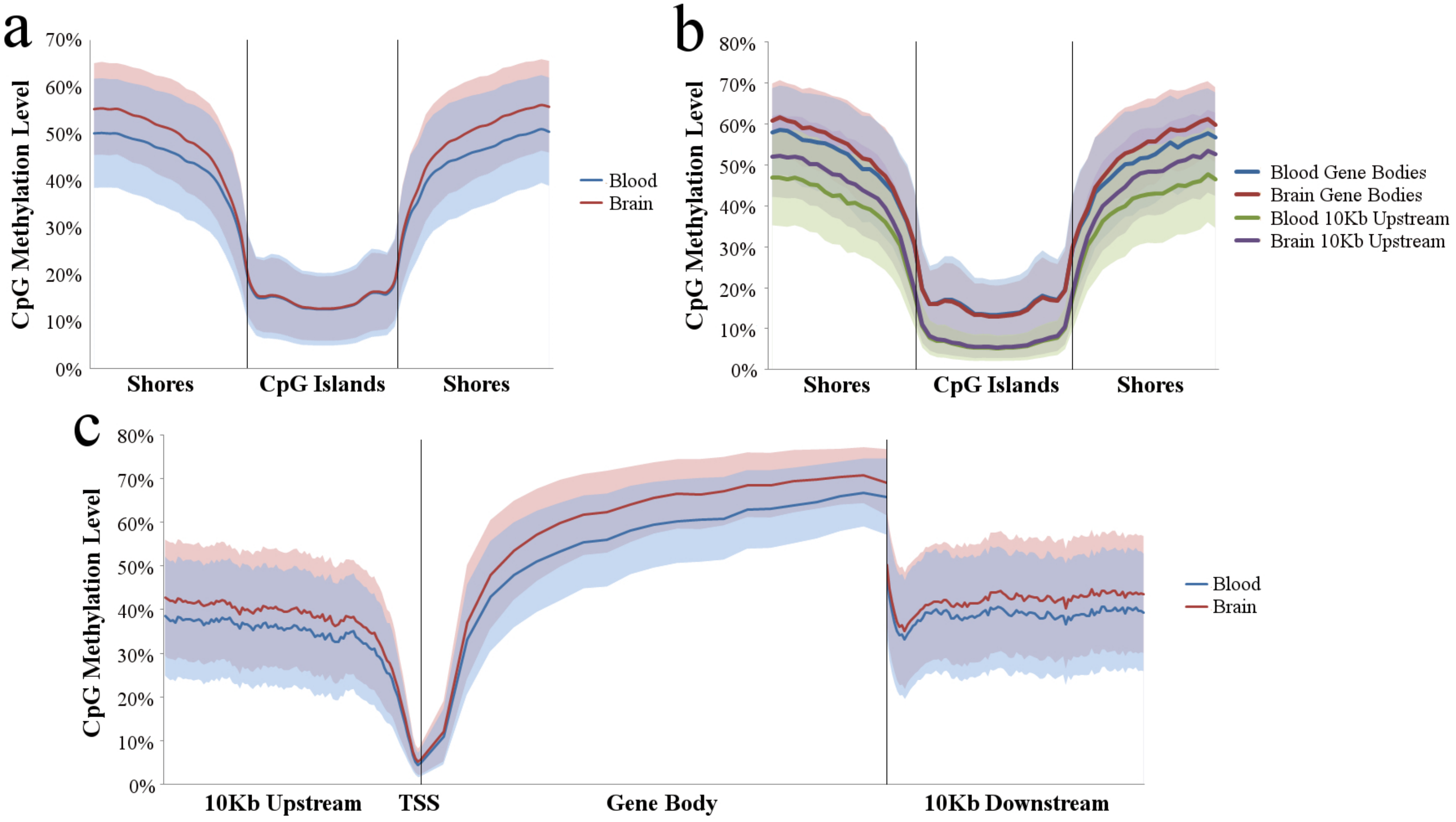

Supplementary Figure 3. CpG methylation patterns across genomic features. CpG methylation profile of (a) CpG islands and shores, (b) CpG islands and shores within gene bodies and up to 10kb upstream of transcription start sites, and (c) gene bodies, including 10kb upstream and downstream. Shaded areas denote variances. Vertical lines denote boundaries of (a, b) CpG islands and (c) gene bodies (annotated transcription start site to transcription termination site).

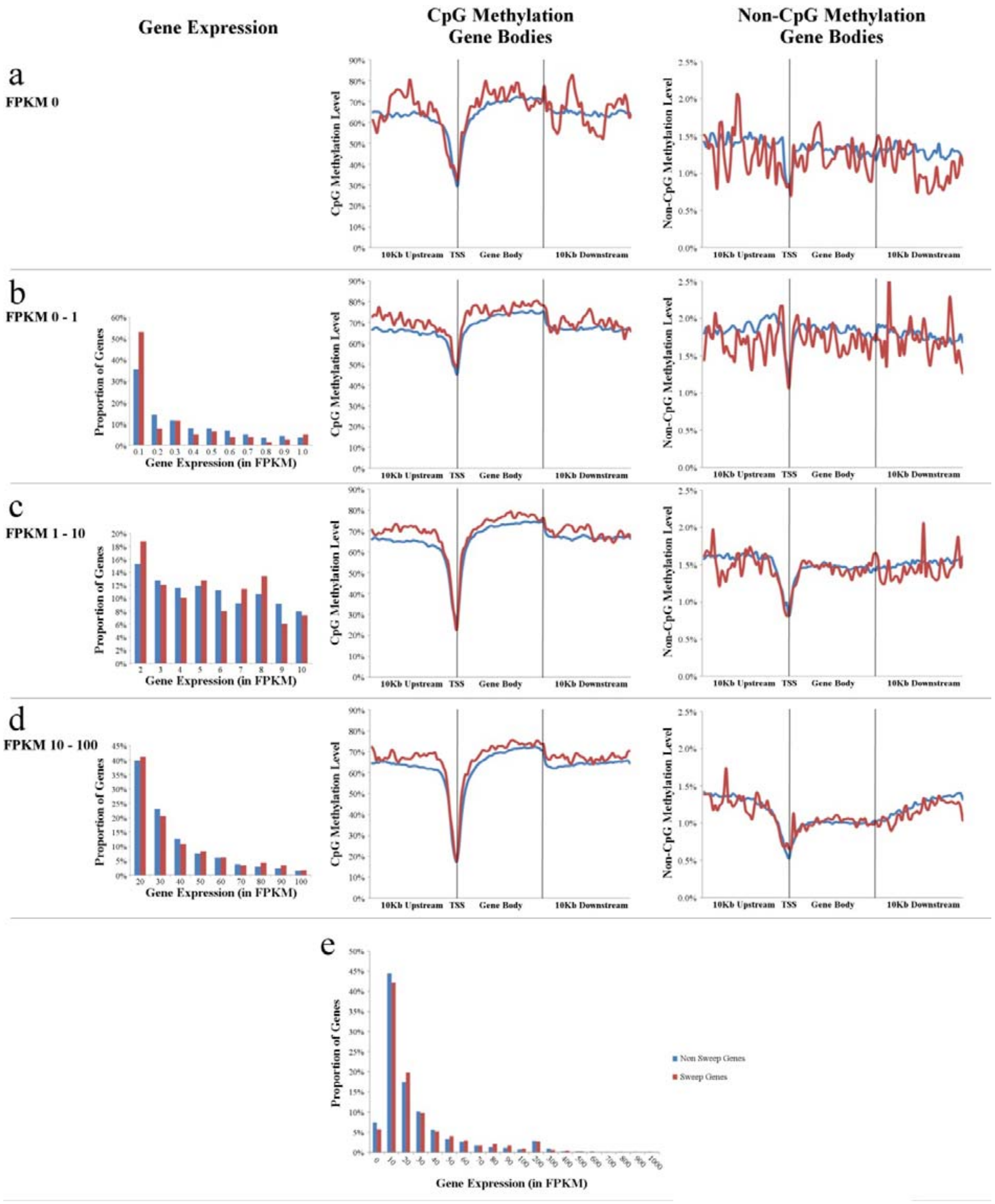

Supplementary Figure 4. Expression and methylation profile of sweep and non sweep genes. Neuronal expression, CpG, and non-CpG methylation profile of sweep and non-sweep genes with (a) no expression, (b) fragments per kilobase of exon per million fragments mapped (FPKM) between 0 - 1, (c) FPKM between 1 - 10, and (d) FPKM between 10 - 100. (e) Expression profile of sweep and non-sweep genes. For example, genes with a FPKM of 10 are expressed at 10 times the rate of genes with a FPKM of 1.

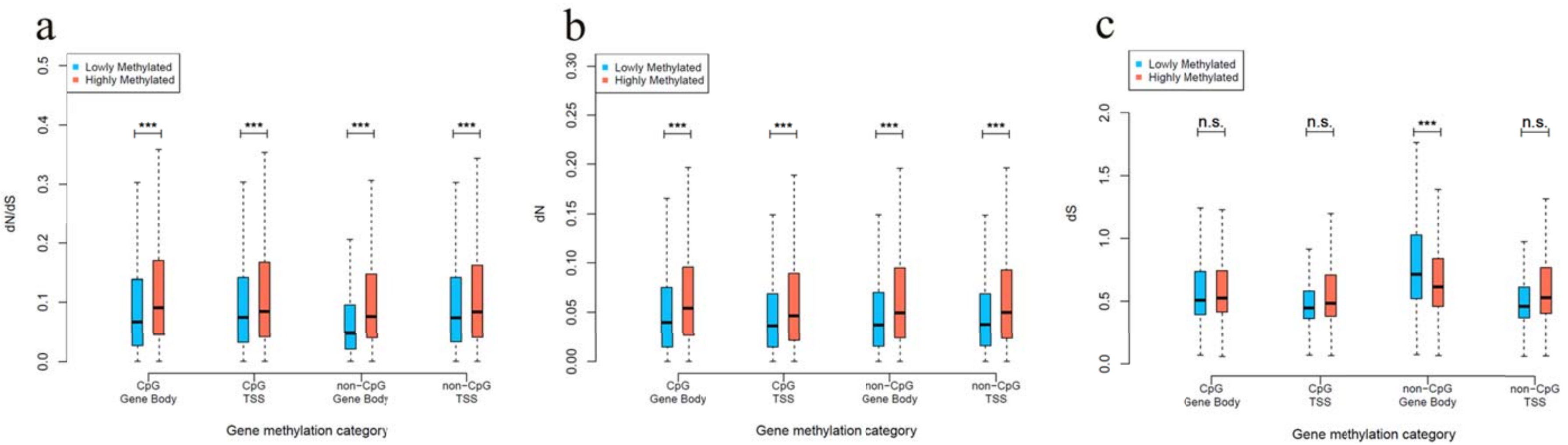

Supplementary Figure 5. DNA methylation levels are associated with evolutionary rates. (a) dN/dS (nonsynonymous substitution rate devided by synonymous substitution rate), (b) dN (nonsynonymous substitution rate), and (c) dS (synonymous substitution rate) values of the lower (lowly methylated) and upper (highly methylated) quartiles of genes in the brain. Four methylation categories with following sample sizes were considered: CpG gene body methylation (1712 lowly and 1618 highly methylated genes), CpG methylation at transcription start sites (2023 versus 2040), non-CpG gene body methylation (972 versus 918) and non-CpG metahylation at transcription start sites (1673 versus 1686). Significance was addressed with a one-sided Mann-Whitney-U-Test). *** P<0.001, n.s. = not significant; the box in a box plot represents the range between upper and lower quartiles, the solid horizontal line denotes the median, and the whiskers show the most extreme data point, which is no more than 1.5 times the length of the box away from the box..

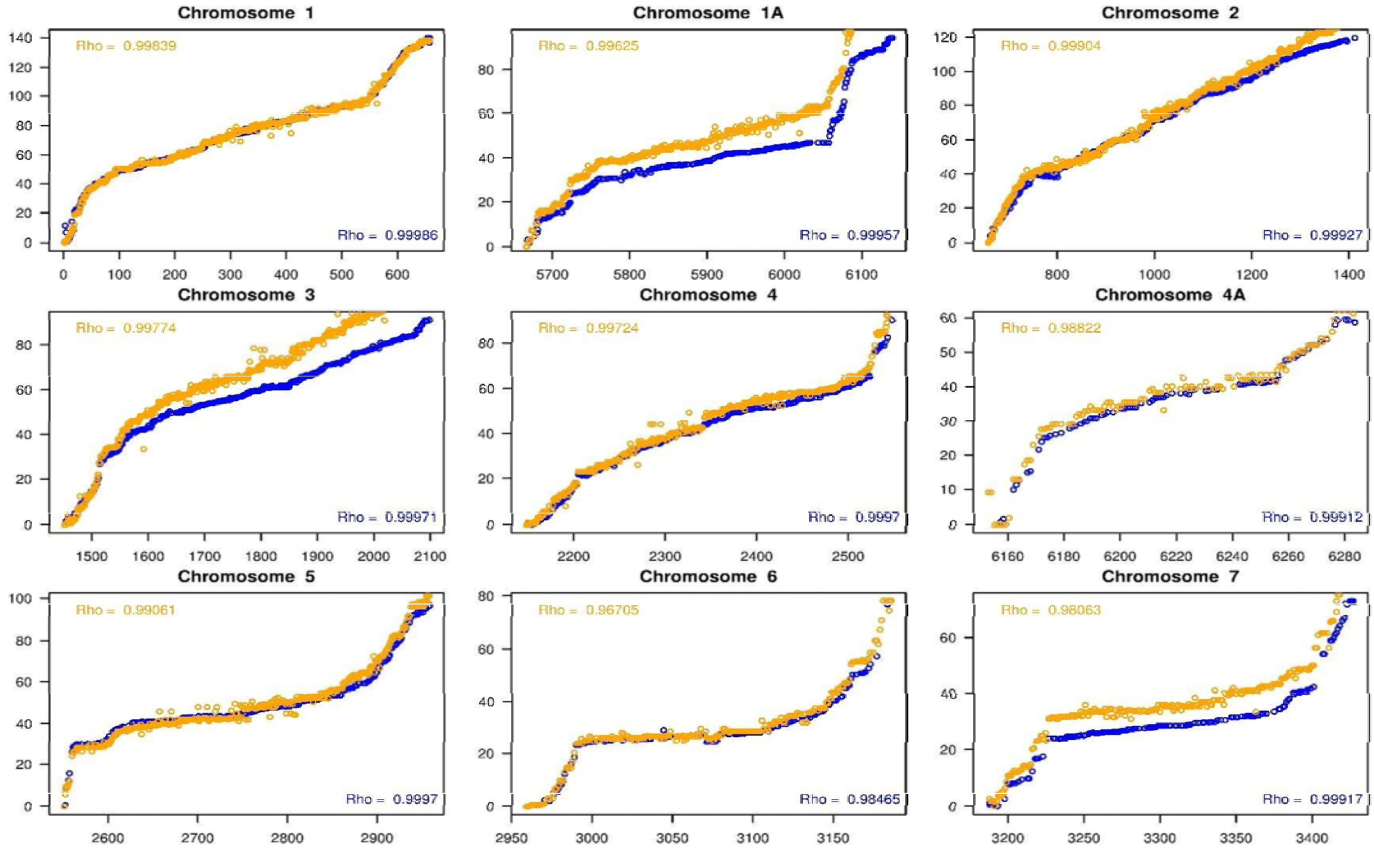
Chromosome 1
Rho = 0.99839
Rho = 0.99986
Chromosome 1A
Rho = 0.99625
Rho = 0.99957
Chromosome 2
Rho = 0.99904
Rho = 0.99927
Chromosome 3
Rho = 0.99774
Rho = 0.99971
Chromosome 4
Rho = 0.99724
Rho = 0.9997
Chromosome 4A
Rho = 0.98822
Rho = 0.99912
Chromosome 5
Rho = 0.99061
Rho = 0.9997
Chromosome 6
Rho = 0.96705
Rho = 0.98465
Chromosome 7
Rho = 0.98063
Rho = 0.99917

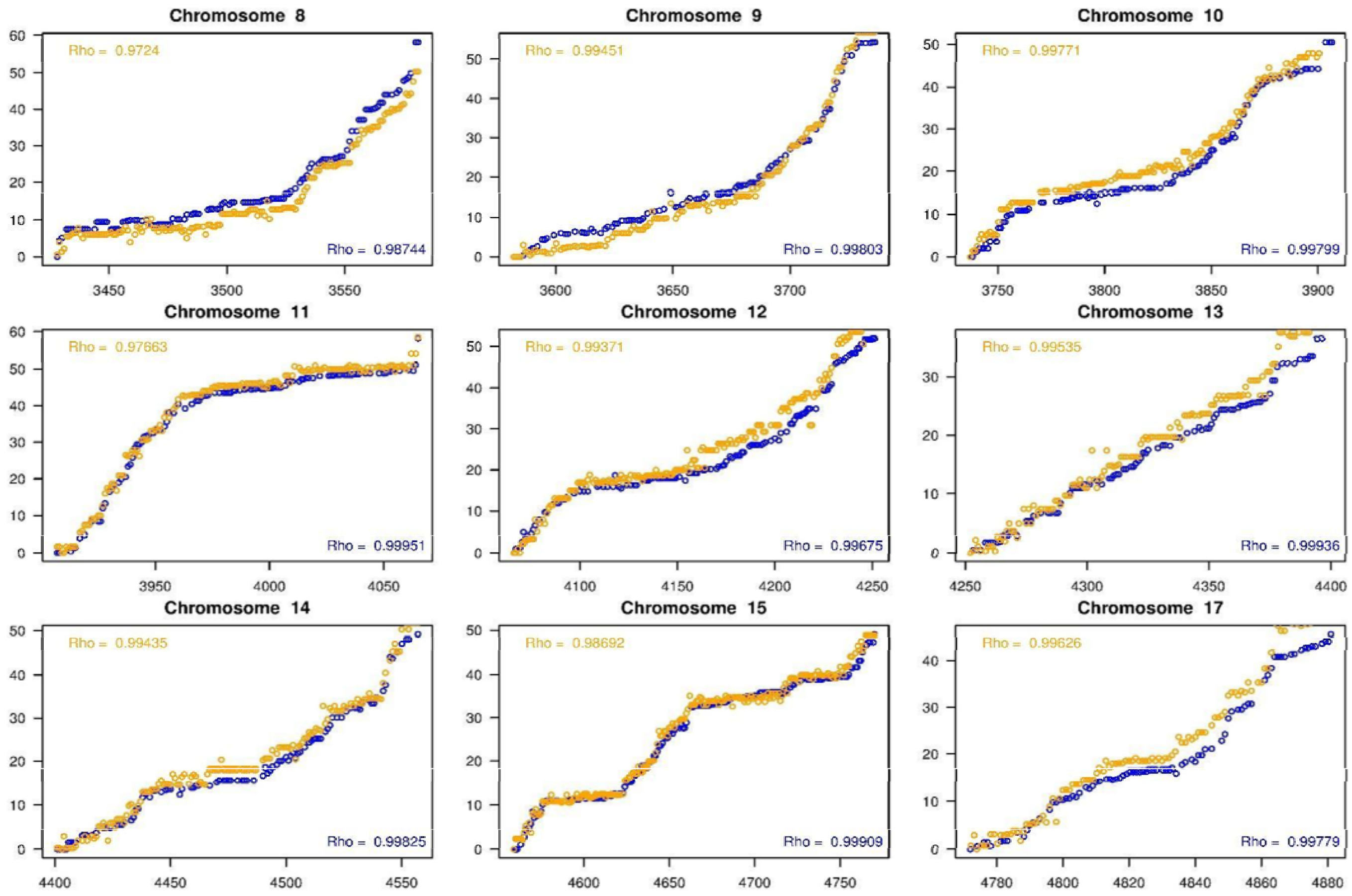
Chromosome 8
Rho = 0.9724
Rho = 0.98744
Chromosome 9
Rho = 0.99451
Rho = 0.99803
Chromosome 10
Rho = 0.99771
Rho = 0.99799
Chromosome 11
Rho = 0.97663
Rho = 0.99951
Chromosome 12
Rho = 0.99371
Rho = 0.99675
Chromosome 13
Rho = 0.99535
Rho = 0.99936
Chromosome 14
Rho = 0.99435
Rho = 0.99825
Chromosome 15
Rho = 0.98692
Rho = 0.99909
Chromosome 17
Rho = 0.99626
Rho = 0.99779

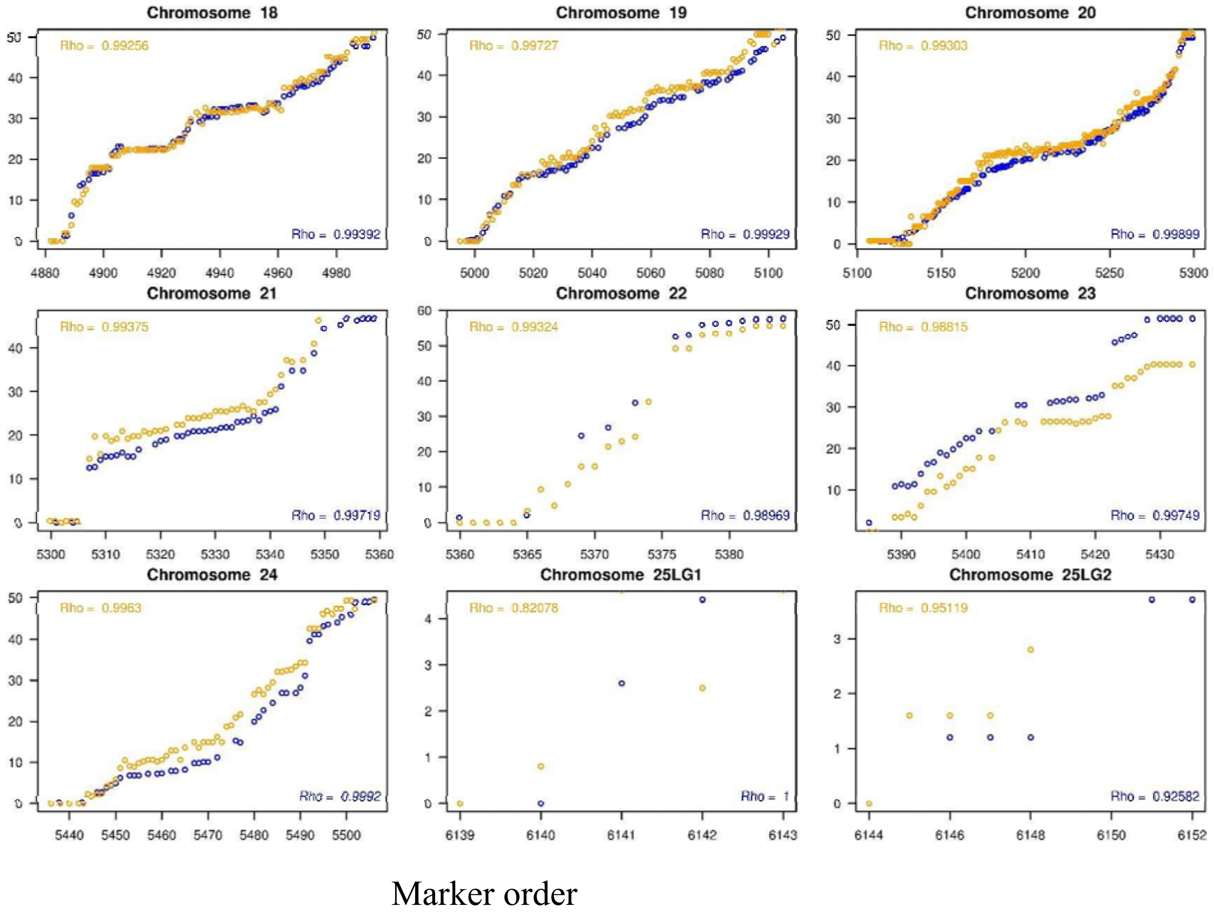

Supplementary Figure 6. Comparison of marker order between the genome assembly and two linkage maps. Each point represents a SNP that was on the NL and UK linkage maps and which could be placed on the assembly scaffold by BLAT. Orange symbols represent the Netherlands map and blue symbols the UK map. Spearman rank correlation coefficients (Rho) between assembly order and linkage map order are indicated and were typically >0.99. X-axis is marker order (units are marker number from 1 to n, where n is number of markers) on the genome assembly. Y-axis is position in cM on the linkage map.

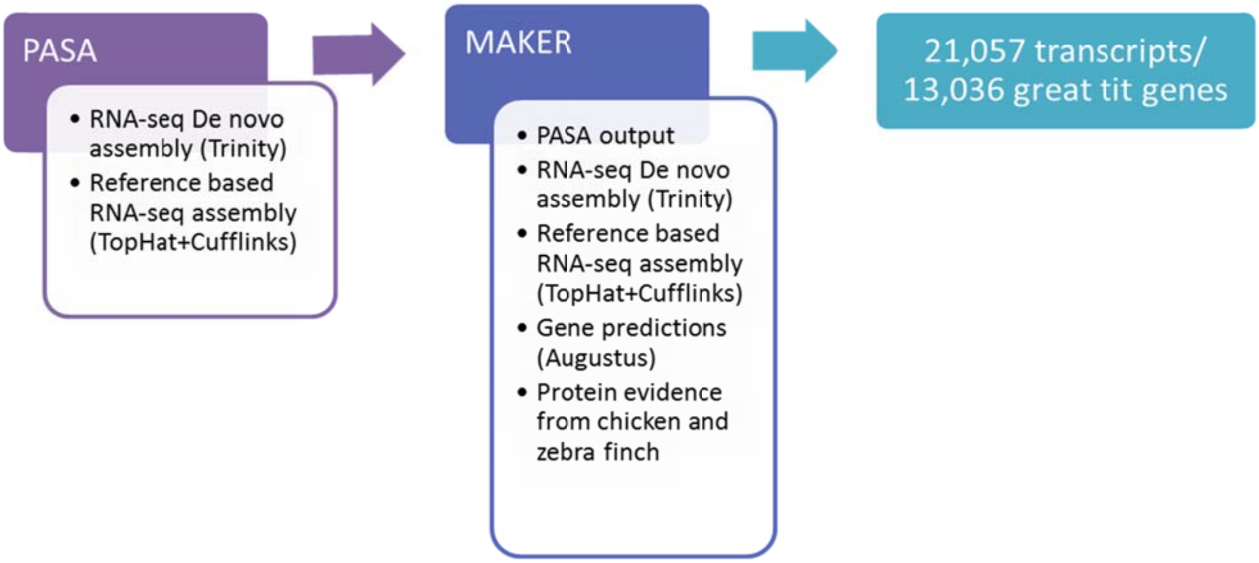

Supplementary Figure 7. Great tit genome annotation pipeline.

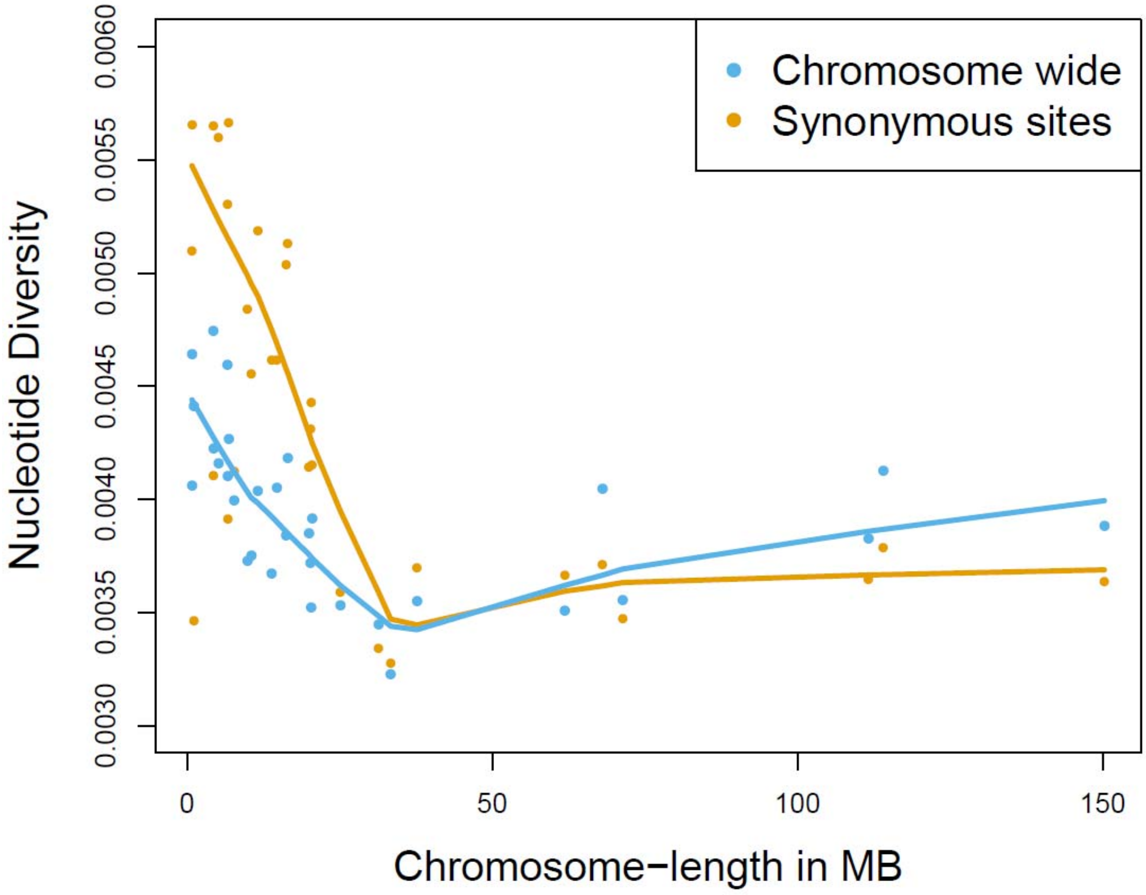

Supplementary Figure 8. Chromosome wide average of nucleotide diversity estimated for synonymous sites and all sites as a function of chromosome size. The solid lines denote local regressions.

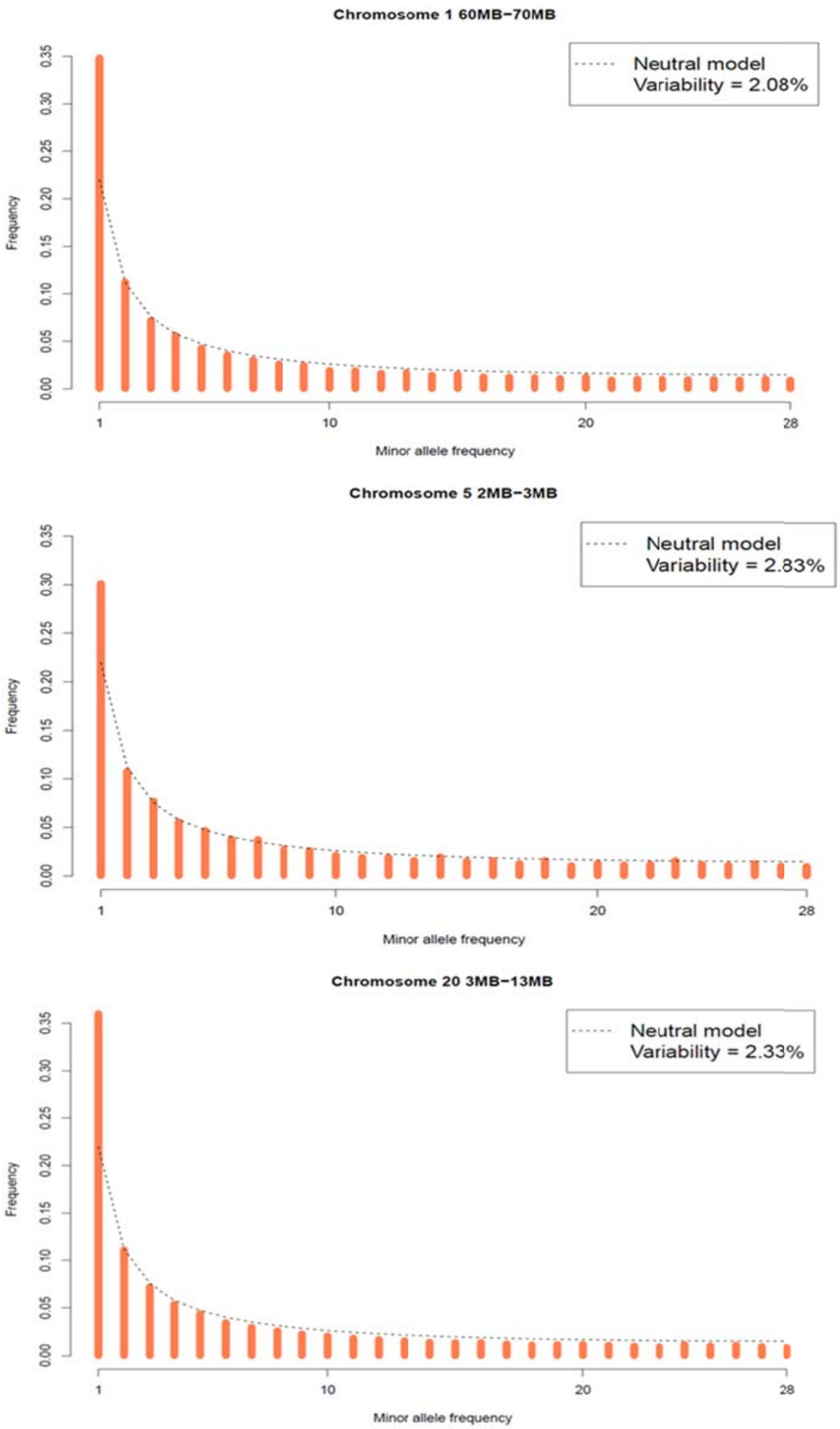

Supplementary Figure 9. Folded site frequency spectra (SFS) derived for regions of chromosomes 1, 5 and 20. Variability denotes the proportion of polymorphic sites and the neutral model denotes the expected site frequency under the neutral theory.

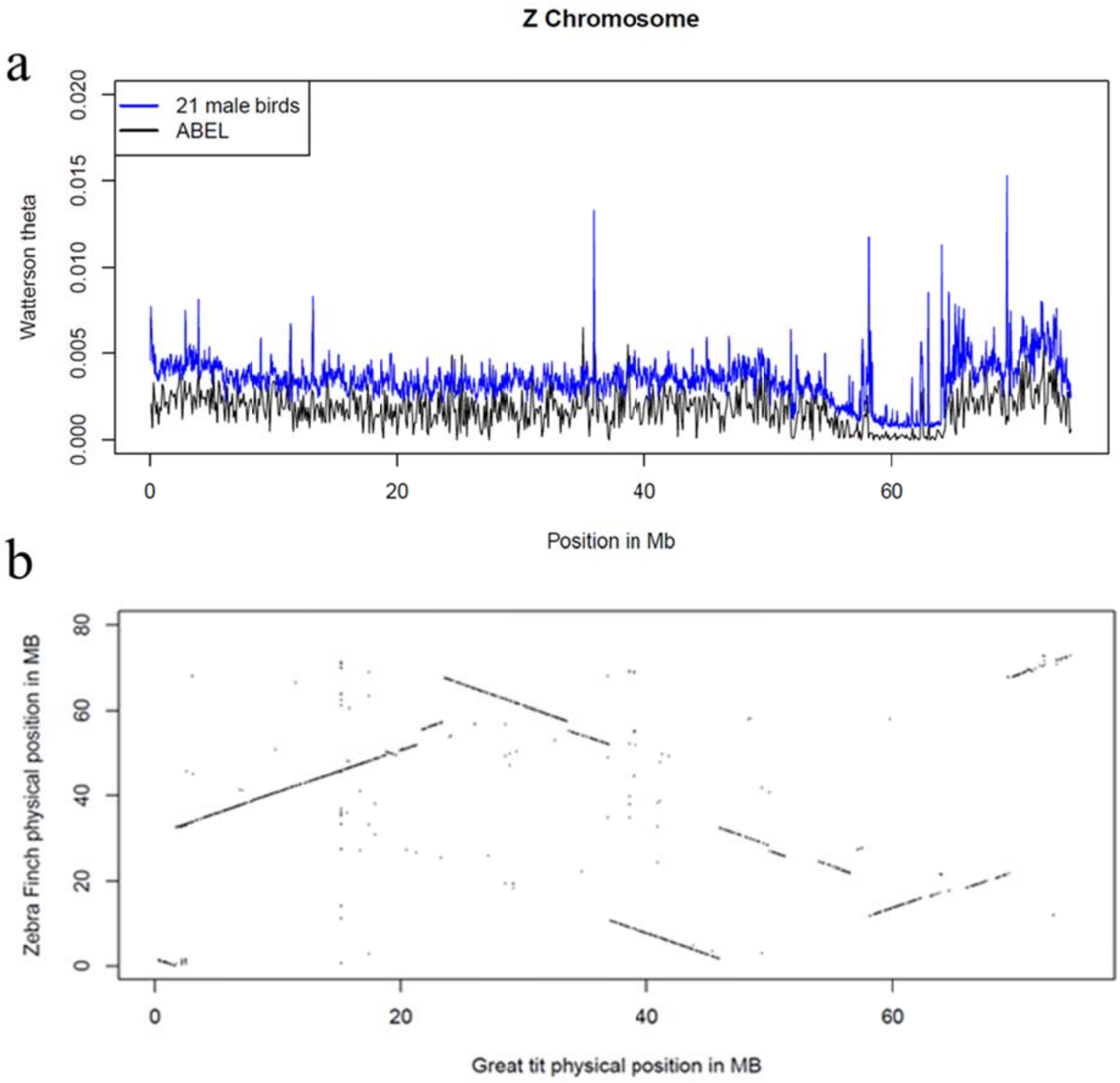

Supplementary Figure 10. Diversity and rearrangements of great tit Z chromosome. (a) Watterson's Θ (a measurement of nucleotide diversity) measured in a sliding window of 10 kB for the Z-chromosome. Diversity for 21 male birds was obtained from the ANGSD pipeline. (b) Chromosome synteny of the zebra finch Z chromosome and the great tit Z chromosome illustrating substantial intrachromosomal rearrangements since the split of the two species.

Supplementary Methods

Study species

Great tits are common, widely distributed, cavity nesting passerines. The range of this super-species spans Europe across Asia to the Pacific, including populations in the Middle East, and North West Africa. From this distribution area four species groups have been identified: *majo*r (whole Europe, Siberia and northwest Africa), *cinereus* (from Iran to India and southeast Asia), *minor* (China, Japan and eastern Russia) and *bokharensis* (central Asia) [4,5]. Most studies have concentrated on the northwest European *Parus major* subspecies and our sequenced bird belongs to this subspecies.

The major subspecies has very distinctive black-yellow-white plumage. The males have brighter colouration and they are larger than females. The great tits living in north-west Europe experience variable environmental conditions which affects their ecology. Great tits are insectivorous during the summer and when the number of insects drops down during the winter they partly switch to seeds and berries. In addition they are regarded as a partial migrant species and during the breeding season they form monogamous pairs and they are territorial. After the breeding season great tits live in flocks which can even include other species.

In their natural habitat they rely on cavities in hardwood trees for nesting. However, they also readily breed in artificial nest boxes and this makes it possible for detailed life history data to be collected for entire populations. This has also allowed them to colonise human environments where nest boxes, other human created cavities, and supplemental feeding support populations. As a result of their abundance and ready use of nest boxes, great tit populations have been intensively studied across their range; some ongoing studies have now collected data on tens of thousands of individuals spanning 60 years. As a result they are now one of the most widely studied species in ecology and evolution.

Genome sequencing and assembly

Sample preparation

The great tit used for this study originated from a captive population artificially selected for four generations for avian personality[6]. The parental generation was taken from two natural populations as 10-day old nestlings and hand reared until independence in the lab. The birds were selected on the basis of their performance in two behavioural tests. Pairs were formed in such a way that there was no inbreeding in the fourth generation. Eggs were laid in an aviary of 2 x 4 x 2.5 m and when females started incubating the whole clutch was transferred to a foster nest in a natural population. The offspring hatching from these eggs then were transported to the lab at day 10 after hatching and hand reared.

The reference great tit was anesthetized using Isoflurane and medical oxygen and euthanized by subtracting all blood from the carotid artery under protocol number CTE-0705 Adendum I, from the Animal Experiment Committee from the Royal Netherlands Academy of Sciences (DEC-KNAW). DNA was isolated from blood samples using the Puregene system (Gentra, USA). The bird was subsequently dissected and all organs were stored separately and snap-frozen at -80 and stored in RNA later. RNA was extracted from eight tissues (bone marrow, homogenized half of the brain, breast filet, higher intestine, kidney, liver, lung and testis) and RNA isolation was performed on 25 mg thawed tissue segments using the miRNeasy mini kit from Qiagen, following the protocol of the manufacturer. For library preparation we used the standard Illumina TruSeqRNAseq protocol using a different barcoded adapters (Illumina) for each of the samples.

De Novo RNA assembly using Trinity

The combined 1 billion reads from all eight tissues were simultaneously de novo assembled using the Trinity software package[7] version r2013-02-25. RNAseq data from the different tissues were combined for the following reasons (1) This would result in one set of predicted gene models with uniform transcript names across the data set and (2) this would also allow better assembly of transcripts present at low levels in multiple tissues. Because of the high depth of the RNAseq data, we first normalized the data. We noticed that due to the high coverage, the data contains a substantial number of retained introns,

resulting in an unrealistically large number of alternative transcripts and unrealistically large average size of the transcripts. We therefore extensively explored different settings for min (2-40) and max (100-1000 (or unlimit)) to find the optimum settings for these parameters to exclude retained intronswhile minimizing the exclusion of rare transcripts. The most optimal settings for our data and the settings used for the final de novo assembly were: --min_kmer_cov 15; --max_cov 400 and --PARALLEL_STATS. Following the normalization, the Trinity assembly was subsequently run using the Trinity default settings. Using these settings, we obtained a total of 101,289 assembled transcripts ranging in size from 201 to 16,061 bp and with an average size of 1,335 bp.

Reference based RNA assembly using Tophat/Cufflinks

The normalized RNAseq data was also analysed with Tophat version v2.0.10[8] (Bowtie v2.1.0[9]) using the following settings: number of hits allowed: 20 (default); inner mate distance 50bp, std inner mate distance 150bp, optimizing mapping accuracy with the "--read-realign-edit-dist" option. Subsequently the tophat alignment output was analysed with Cufflinks version 2.2.0[10] including the following settings: --overlap-radius 5 ('distance to split genes' - to avoid merging of adjacent genes; the default is 50 but we observed that this occasionally led to merging of genes that are close together).

Functional annotation and repeat/RNA masking

Functional annotation was done by using InterProScan version 5.4-47.0[11]. REPEATMASKER v. 4.0.3 (http://www.repeatmasker.org/) with option -gccalc and query species “aves” was used to mask the repeats in the assembly. In total 6.55 % of the genome was covered (Supplementary Data 8.). In addition to repeats, tRNAs were identified by using tRNASCAN-SE version 1.3.1. with default settings[12].

Comparison of Maker gene predictions with *Parus major* 454 sequence contigs

To obtain further insight in our gene annotation, we compared the Maker transcripts to a previously reported[13] large *Parus major* transcriptome sequencing effort using 454 sequence technology. In that study also RNA from eight different tissues was used. Five of the tissues used in that study were the same

as in our study (brain, kidney, liver, muscle and testis) while 3 were different (heart, pancreas and skin). A major difference between the two studies is that in the 454 study, the sequencing libraries were normalized. A total of 4.6 million 454 sequences (average size; 302 bp) were assembled into 95,979 contigs. We aligned the 454 contigs against all transcript identified by Maker using blastn. In total, 43,049 contigs did align against 15,013 Maker transcripts representing 10,873 different genes. As expected, this number is considerably higher than that for chicken and zebrafinch (34,844 and 33,574 respectively[13]). We repeated the alignment of the 454 contigs against the chicken and zebrafinch transcript databases used in our study and obtained similar results (32,177 and 36,684 respectively).

The majority of the 454 contigs (48,999) did not align to any of the transcript sequence of these three birds. Interestingly a large proportion (43%) of these 48,999 contigs did align to intronic sequences of the annotated great tit genes. These results suggest that a large proportion of the 454 contigs represent artefacts (e.g. retained introns) and other non-coding transcripts like long noncoding RNAs (lncRNA), most likely as a result of the normalization of the cDNA libraries. Around 8.4 % (3931) of the aligned 454 contigs did not align to any of the great tit Maker transcripts although 1106 of these did align to intronic sequences of the annotated great tit genes.

Preparing samples for whole-genome bisulfite sequencing

Whole-genome bisulfite sequencing was conducted on the same individual. DNA was isolated from whole blood using a GentraPuregene Kit, Qiagen, USA. The brain sample was incubated overnight at 55 °C in 750 µl Cell Lysis Solution (GentraPuregene Kit, Qiagen, USA), with 20 µl proteinase K. To remove excess of fat and proteins, 24:1 chloroform:isoamylalcohol was added and mixed until homogeneous, followed by 10 min centrifugation at 12.000x g after which the upper layer was collected. Cell Lysis Solution was added to this upper layer until 500 µl of sample liquid was obtained and total DNA was extracted according to the manufacturer's protocol. DNA was stored in DNA Hydration Solution (Qiagen, USA).

CpG Island Prediction

CpG Islands were predicted using cpgplot of the EMBOSS package (version 6.6.0.0) applying default settings (-window 100 -minlen 200 -minoe 0.6 -minpc 50). CpG shores were defined as 2kb upstream and downstream of islands.

Great tit samples and data generation for resequencing

The resequenced 29 great tit individuals (Supplementary Data 2) were from a wide range of the species distribution (Figure 1); 10 individuals were from the UK Wytham population in Oxford (UK), another 19 birds were sampled from 15 European populations. Each bird was sequenced at ≈10x coverage. 21 birds were males and 8 were females, DNA was extracted from avian blood and quantified to a concentration of 50-200 ng/µl. Samples were treated with Rnase I recombinant, E. coli - 1000U (Lucigen) (VWR 89002-444) at The Genome Institute, Washington University, where they were then sequenced as follows. DNA from each sample was randomly fragmented by nebulization to an average size of ca. 300 bp, and processed by the Illumina DNA sample preparation protocol (Illumina, San Diego, CA), including end-repair, add-tailing, paired-end adaptor ligation and PCR. Paired-end sequencing libraries of each sample were built with an insert size of 300 bp and sequencing was performed on a HiSeq 2000 platform with a read length of 100 bp.

The reliability of SNP data

Tests were carried out to assess the reliability of the SNP calls. First, we calculated nucleotide diversity in 50kb windows using data from the 29 birds (10x coverage), and compared the values to those based on data from the single male individual (reference bird) that was used to assemble the reference genome (30x coverage using the short-insert library). As can be seen in Supplementary Data 9, there is a high level of consistency between diversity estimates based on the 29-bird data set and the reference bird data set (Genome wide Spearman rho=0.69). Second, we compared relative levels of diversity and the site frequency spectra across three genomic regions with different recombination rates[14]: inner sections of chromosome 1 (macrochromosome; low recombination), the tips of chromosome 5 (macrochromosome;

high recombination), and chromosome 20 (microchromosome; high recombination). This choice is to make use of the fact that varying rates of recombination affect the efficacy of natural selection across the great tit genome[15]. As a result, we expect different chromosomes to show different polymorphism patterns because they are subject to different intensity of Hill-Robertson interference (HRI)[16]. As predicted by the HRI theory, observed diversity levels, were higher in regions of high recombination (Supplementary Fig. 8 and 9).

Scans for selective sweeps

To identify targets of positive selection in the great tit genome we used Sweepfinder[17], which uses local deviations of the folded site frequency spectra (SFS) from a reference (e.g. chromosome wide SFS). Since the coverage in our data is relatively low (approx 10x per individual), it is crucial to carefully prepare the SNP calling set. To prepare the input files for Sweepfinder we used ANGSD[18] to call the SNP set with a minimum mapping quality score of 20 and minimum base quality score of 20. We also excluded SNPs for which the coverage was exceeding twice the total coverage of the respective chromosome or less than half of the total coverage on that chromosome. SNPs with coverage in less than 70% of the individuals (42 haplotypes for autosomes and 30 for the Z-chromosome) were excluded as well. Furthermore we excluded the top 1% of SNPs with extreme strand bias and deviation from Hardy-Weinberg-Equilibrium (function SNP filters in ANGSD). We then inferred the allele frequency for each variable position from ANGSD (-doMaf 2) using the reference genome which then was subsequently converted to the input format of Sweepfinder with customized scripts. To infer the ancestral states of each SNP we used maximum parsimony. We obtained whole genome alignments of the reference genome using MAUVE[19]. The used outgroup genomes were ground tit, zebra finch, flycatcher and chicken. Sites that could not be reconstructed with confidence were used as folded in the Sweepfinder analysis.

To speed up computation we run Sweepfinder on 10MB chunks (including additional runs for shifted chunks with 5MB overlap, to identify potential sweeps near cutoffs) for each chromosome. The reference SFS was created for each whole chromosome. The grid size was 100 bp, this corresponds to approx. 10

million sites that were tested. We then extracted the composite likelihood ratio (CLR) values from the runs, if the CLR value from the shifted run of the closest nearby tested position (<=100bp) was higher, this value was used. Sweep hits then were merged to sweep regions when the neighbouring score(s) exceeded a certain threshold, which was set to the top 1% of CLR scores (i.e. approx. 5) and minimum size sweep size of larger than 300bp. For final score for each sweep regions is the sum of CLR scores of the sites of the sweep region.

Sweep regions have then been processed further to extract genes, but we conducted two filter steps before gene extraction. First, sweep regions were excluded that contained too many non-sequenced positions (i.e. more than 10% of N's in the sweep region+2kb flanking region on each side). Second, if individual coverage for the whole sweep region and 2kb flanks exceeded a certain value they were excluded as well. Exclusion criteria were if the individual coverage of the whole sweep region exceeded 2.5 times the average coverage of that individual on the same chromosome or if individual exceeded 3 times the average coverage of the region of all individuals. Genes overlapping the sweep region or 5KB flanking regions on either side of the sweep region were included and the top 3% (=534) of genes were used for the subsequent analysis. We also checked basic test statistic of the remaining sweep regions versus a set of random regions on the same chromosomes of the same sizes. Sweeps tend to have lower diversity and lower Tajima's D (e.g. a classic sweep signal) and they do not show any bias regarding their coverage, base quality or repeat content in comparison to the random set.

Low diversity region of the Z chromosome

To further investigate the low diversity region of the Z chromosome we estimated diversity with the ANGSD pipeline using only the 21 birds for which the gender was known to be male. Additionally, we inferred diversity by calling heterozygous sites from the reference individual with Platypus[20], which was also a male. The low diversity region is located between 58 MB and 64 MB on the Z-chromosome and also coincides with a rearranged block on Z-chromosome relative to the zebra finch Z-chromosome

(Supplementary Fig. 10b). Taken together these analyses allow us to exclude low-coverage as the cause of the low diversity pattern on the Z chromosome.

The consensus SNP set

To construct a consensus dataset with high quality SNPs, we took the intersection of the SNPs that have been called by all four approaches (Supplementary Data 10). Note that this variant set has to be considered highly conservative. For this, we focused on the assembled chromosomes and discarded variants within scaffolds. Furthermore we had data from more than 1600 great tit individuals genotyped on a 650K SNP array (data will be described elsewhere). From the SNP array dataset we extracted high frequency SNP positions (minor allele frequency >20% in the overall population as well as for the 27 birds, 2 were not included on the array) to recalibrate the variant scores from the "Multi" variant calling with GATK[21] and included only variants that passed the SNP call with the recalibrated score (most conservative approach). We also used SNPable (http://lh3lh3.users.sourceforge.net/snpable.shtml) to mask the genome for regions in which short sequencing reads cannot be uniquely mapped with great confidence using standard parameters (l=0.35, r=0.5). SNPs falling into regions of low certainty as defined by SNPable were subsequently excluded from the final dataset. Altogether, the final dataset consisted of ≈ 4.5 million SNPs.

Supplementary References

1. Harris, R. Improved pairwise alignment of genomic DNA. (2007).
2. Jarvis, E. D. *et al.* Whole-genome analyses resolve early branches in the tree of life of modern birds. *Science* **346**, 1320–1331 (2014).
3. Schneider, T.D., Stephens, R.M. Sequence logos: a new way to display consensus sequences. *Nucleic Acids Res.* **18,** 6097–6100 (1990).
4. Gosler, A. *The Great Tit*. (Hamlyn, 1994).
5. Päckert, M. *et al.* The great tit (*Parus major*) - A misclassified ring species. *Biol. J. Linn. Soc.* **86,** 153–174 (2005).

6. Drent, P. J., van Oers, K. & van Noordwijk, A. J. Realized heritability of personalities in the great tit (*Parus major*). *Proc. R. Soc. B Biol. Sci.* **270,** 45–51 (2003).
7. Grabherr, M. G. *et al.* Full-length transcriptome assembly from RNA-Seq data without a reference genome. *Nat. Biotechnol.* **29,** 644–652 (2011).
8. Kim, D. *et al.* TopHat2: accurate alignment of transcriptomes in the presence of insertions, deletions and gene fusions. *Genome Biol.* **14,** R36 (2013).
9. Langmead, B., Trapnell, C., Pop, M. & Salzberg, S. L. Ultrafast and memory-efficient alignment of short DNA sequences to the human genome. *Genome Biol.* **10,** R25 (2009).
10. Trapnell, C. *et al.* Transcript assembly and quantification by RNA-Seq reveals unannotated transcripts and isoform switching during cell differentiation. *Nat. Biotechnol.* **28,** 511–515 (2010).
11. Zdobnov, E. M. & Apweiler, R. InterProScan - an integration platform for the signature-recognition methods in InterPro. *Bioinformatics* **17,** 847–848 (2001).
12. Lowe, T. M. & Eddy, S. R. tRNAscan-SE: A program for improved detection of transfer RNA genes in genomic sequence. *Nucleic Acids Res.* **25,** 955–964 (1997).
13. Santure. A., Gratten, J., Mossman, J.A., Sheldon, B.C. & Slate, J. Characterization of the transcriptome of a wild great tit *Parus major* population by next generation sequencing. *BMC Genomics* **12**, 283 (2011).
14. Van Oers, K. *et al.* Replicated high-density genetic maps of two great tit populations reveal fine-scale genomic departures from sex-equal recombination rates. *Heredity* **112,** 307–316 (2014).
15. Gossmann, T. I., Santure, A. W., Sheldon, B. C., Slate, J. & Zeng, K. Highly variable recombinational landscape modulates efficacy of natural selection in birds. *Genome Biol. Evol.***6**, 2061-2075 (2014)
16. Cutter, A. D. & Payseur, B. a. Genomic signatures of selection at linked sites: unifying the disparity among species. *Nat. Rev. Genet.* **14,** 262–274 (2013).
17. Nielsen, R. et al. Genomic scans for selective sweeps using SNP data. *Genome Res.* **15**, 1566–1575 (2005).

18. Korneliussen, T., Albrechtsen, A. & Nielsen, R. ANGSD: Analysis of Next Generation Sequencing Data. *BMC Bioinformatics* **15**, (2014).

19. Darling, A. E., Mau, B. & Perna, N. T. progressiveMauve: Multiple Genome Alignment with Gene Gain, Loss and Rearrangement. *PLoS One* **5**, e11147 (2010).

20. Rimmer, A. et al. Integrating mapping-, assembly- and haplotype-based approaches for calling variants in clinical sequencing applications. *Nat. Genet.* **46**, 912–918 (2014).

21. DePristo, M. A. et al. A framework for variation discovery and genotyping using next-generation DNA sequencing data. *Nat. Genet.* **43**, 491–498 (2011).
